# Adolescent Exploratory Strategies and Behavioral Types in the Multivariate Concentric Square Field^TM^ Test

**DOI:** 10.3389/fnbeh.2019.00041

**Published:** 2019-03-04

**Authors:** Stina Lundberg, Cecilia Högman, Erika Roman

**Affiliations:** Neuropharmacology, Addiction and Behavior, Department of Pharmaceutical Biosciences, Uppsala University, Uppsala, Sweden

**Keywords:** behavior, elevated plus maze, open field, phenotyping, risk taking, social play, validation, Wistar rat

## Abstract

Adolescence is an important developmental phase with extensive changes in behavior due to remodeling of the brain and hormonal systems. Validation of animal behavioral tests in this age group is therefore of importance as differences to adult behavior are often not clarified. The aim of the present study was to investigate adolescent behavior in the multivariate concentric square field^TM^ (MCSF) test and its relationship to other common behavioral tests as well as to a literature dataset of adult animals. Sixty adolescent male Wistar rats were tested in the MCSF and one of four reference tests; the elevated plus maze, the open field with or without start box, or the social play behavior test. Additionally, 12 animals were tested twice in the MCSF. When analyzing the first encounter with the MCSF test, a distinct grouping of the individuals into three *behavioral types* was observed. Approximately 20% of the animals had high levels of activity and an additional 20% had high levels of shelter seeking-behavior, these groups composed the outlying behavioral types named Explorers and Shelter seekers, respectively, which were distinct from the Main type of animals. When tested in the MCSF for a second time, the adolescent animals showed a recollection of the arena as they changed their behavior in relation to the first encounter. When comparing the MCSF performance to the reference tests, a relationship was found between the MCSF and the other behavioral test entailing forced exploration, while no relationship was found between the MCSF and social play. The adolescent behavioral profile was characterized by decreased risk assessment and a different activity profile than adults. In conclusion, the MCSF test is useful for profiling adolescent rats but the behavioral interpretation differs from that of adults due to differences in behavioral manifestation during adolescence and the presence of natural subgroups. Adolescent exploration shows a relationship across tests, but the MCSF gives more information than any of the other behavioral tests based on forced exploration. Further studies into the neurobiology behind the behavioral types and how different manipulations affect the distribution into the behavioral types are of interest.

## Introduction

Adolescence is the developmental stage between child- and adulthood and is of interest as it is a period of substantial changes in brain structure and function as well as in behavior. It is also a period when many psychiatric disorders, including mood and eating disorders, addiction and schizophrenia, first manifests (Sturman and Moghaddam, 2011; Crews et al., 2016). Adolescents across mammalian species show increased social behaviors, increased risk taking, and more sensation- and novelty seeking than both younger and older individuals (Doremus-Fitzwater et al., 2010; Sturman and Moghaddam, 2011). This is thought to facilitate the transition from immature juvenile to independent adult and aid in the search for new territory, food sources, and mates (Yurgelun-Todd, 2007). The brain alterations during adolescence are extensive; almost all neurotransmitter systems as well as the white and gray matter are still developing (Ernst et al., 2006; Casey et al., 2008; Sturman and Moghaddam, 2011; Crews et al., 2016). Different parts of the brain mature in different rates, accounting for the cognitive and behavioral inconsistencies during adolescence. Imbalance in the risk-reward system make adolescents more impulsive and reckless in the search for sensation, reward, and novelty, and difficulties to integrate emotional information can lead to poor decision-making and an elevated emotional state (Ernst et al., 2006; Doremus-Fitzwater et al., 2010; Sturman and Moghaddam, 2011; Crews et al., 2016).

Behavioral studies of laboratory animals are used in research for many purposes, from evaluating how a certain genetic variant impact the behavioral phenotype to evaluating pharmaceutical candidates and animal models of human conditions (Hånell and Marklund, 2014; Brown and Bolivar, 2018). A common feature of many of the behavioral tests commonly used today is that only one behavior or type of behavior is studied per test and for some behavioral tests their popularity seems to stem from convenience rather than theoretical reasons (Schellinck et al., 2010; Fonio et al., 2012; Hånell and Marklund, 2014; Garner et al., 2017; Kafkafi et al., 2018). If a wider behavioral characterization is desired, a battery of different tests is often used (Blanchard et al., 1998; Budde et al., 2013; Trent and Menard, 2013; Hånell and Marklund, 2014). A problem with this approach is that there can be carry-over effects between the tests so that the experience gained in one test influence the performance in subsequent ones (McIlwain et al., 2001; Paylor et al., 2006; Blokland et al., 2012). This problem can be avoided with the use of more diverse behavioral tests that give the animal a choice between different types of environments and thereby the ability to express a broader behavioral repertoire (Garner, 2014; Stewart and Kalueff, 2015). Moreover, the fact that experimenter handling of animals is a large source of variation (Deacon, 2006; Schellinck et al., 2010; Garner, 2014) favors the use of one comprehensive test rather than multiple testing in behavioral test batteries.

The multivariate concentric square field^TM^ (MCSF) test is a behavioral test developed to study rodent behavior in a diverse, multivariate setting, emulating the diversity in the natural environment (Meyerson et al., 2006). The outline of the MCSF is based on an ethoexperimental foundation, i.e., the design is ethologically founded with regard to what rodents naturally associate with exploration, risk, and shelter. The features of the MCSF test are based on the innate behavior of rodents and are similar to other commonly used behavioral tests: the open field (OF) test corresponds to the open center of the MCSF; and the elevated plus maze (EPM), where the closed arms of the EPM corresponds to the corridors in the MCSF, and the open arms of the EPM are mimicked by the bridge construction in the MCSF. Moreover, the lighting conditions in the MCSF test are inspired by the commonly used light/dark box (LDB) test so that the bridge is brightly illuminated whereas the rest of the arena has more dimmed light conditions. Additionally, the MCSF includes a dark enclosure, giving the animal the possibility to seek shelter. These varied conditions of the MCSF arena give the animal a choice between different areas to explore and thereby the chance to express a range of behaviors. As a result, a session in the MCSF can give an extensive behavioral profile including information about activity-, risk-, and shelter-associated behaviors (Meyerson et al., 2006, 2013; Roman et al., 2012) which are evolutionary conserved. The MCSF was initially developed to study adult mice and rats (Roos et al., 2003; Augustsson and Meyerson, 2004; Meyerson et al., 2006) and has recently been used for behavioral profiling of adolescent rats after different early environmental challenges (Palm et al., 2013; Berardo et al., 2016; Wille-Bille et al., 2017, 2018).

Validation of scientific methods is of the highest importance, as without valid methods accurate conclusions cannot be drawn. Animal behavioral tests can be difficult to validate (Schellinck et al., 2010; Garner et al., 2017) because there is no direct way to know what animals’ behaviors mean; we need to rely on ethological theories, evolutionary conserved mechanisms, and use tools like pharmaceuticals that affect behaviors to guide behavioral interpretations (Nestler and Hyman, 2010). Additionally, it is difficult to thoroughly standardize behavioral tests and unwanted variance can thus be introduced between different labs and operators affecting the reproducibility (Schellinck et al., 2010; Fonio et al., 2012). The need for in-depth knowledge of factors influencing behavioral outcomes when interpreting results from behavioral experiments (Schellinck et al., 2010; Garner et al., 2017) and the question to standardize or not (Voelkl et al., 2018) may, in part, lie behind the reported poor validation and reproducibility in the field of behavioral neuroscience (Schellinck et al., 2010; Garner et al., 2017).

The present study is part of the ongoing process to thoroughly validate and describe behavior in the MCSF test. The specific aim was to characterize adolescent behavioral strategies in the MCSF test in animals without prior treatments. Furthermore, the aim was also to investigate how the behavior in the multivariate arena relates to the performance in other commonly used forced exploration tests: the EPM, OF test, and a modified OF that also provides a sheltered area, i.e., the OF with start box, as well as to social behavior in the social play behavior (SPB) test. The hypothesis was that the MCSF test would encompass behaviors expressed in several of the other, more univariate, tests and thus, that testing in the MCSF would give a comprehensive picture of the overall behavioral profile in one test.

## Materials and Methods

### Animals, Housing, and General Procedure

**FIGURE 1:**
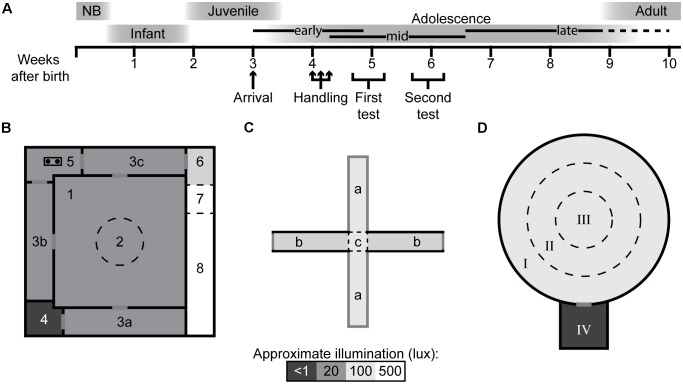
Experimental outline and behavioral test arenas with defined zones. **(A)** Experimental outline with rat developmental stages and age in weeks (NB, newborn) (Tirelli et al., 2003; Alberts, 2005; Spear, 2015). Schematic layout of the **(B)** multivariate concentric square field^TM^ (MCSF), **(C)** elevated plus maze (EPM), and **(D)** open field (OF) (with start box) arenas. The arenas are divided into zones by walls (solid, black lines) or imagined boundaries (dashed lines). MCSF zones: (1) center, (2) central circle (CTRCI), (3a–c) corridors, (4) dark corner room (DCR), (5) hurdle, (6) slope, (7) bridge entrance, and (8) bridge. EPM zones: (a) open arms, (b) closed arms, and (c) central square (CTRSQ). OF zones: (I) outer circle (OC), (II) inner circle (IC), (III) center (CTROF) and, when used, (IV) start box. The saturation level in the figures reflects the approximate illumination levels in the arenas.

The experimental outline is summarized in Figure 1A together with the approximate ages of all procedures and their place in the rat development. In this study, 72 outbred male Wistar rats (RccHan^TM^:WI, Harlan Laboratories B.V., now Envigo, Horst, Netherlands) were used, arriving at the animal facility at approximately 3 weeks of age. The animals arrived in three separate batches of 24 animals over the span of 2 months. The animals were housed in groups of three to four in transparent type IV cages (59 × 38 × 20 cm) with raised cage lids, and pelleted food (Type R36, Lantmännen, Kimstad, Sweden) and tap water *ad libitum*. The bedding consisted of wood-chip and two paper sheets (40 × 60 cm, Cellstoff, Papyrus) per week as enrichment. The animal room was kept in constant temperature (22 ± 1°C) and humidity (50 ± 10%) on a reversed, 12 h light/dark cycle (lights off at 07:00) with 30 min of dusk/dawn. The test rooms were kept at similar conditions as the animal room and all rooms had a masking background noise to minimize unexpected sounds that could disturb the animals.

The animals were left undisturbed for 1 week to acclimatize to the animal facility after which they were individually marked by ear notching and subjected to three consecutive days of handling, weighing, and adaptation to a transportation bucket that later was used for transportation of individual rats from the home cage to the test arenas. All handling and testing were performed during the dark period of the light/dark cycle by female experimenters.

At approximately 5 weeks of age (12–15 days after arrival) the animals performed their first behavioral test and a week after that, at week 6 of age, they performed the second behavioral test. Thirty-six of the animals were first tested in the EPM test, OF test, or the OF test with start box (*n* = 12/group) followed by the MCSF test. Twelve animals were tested repeatedly in the MCSF test and 24 animals performed the SPB and MCSF tests in counterbalanced order. Directly after each behavioral test the animals were weighed and their body weight recorded.

The experimental protocol was approved by the Uppsala Animal Ethical Committee (C27/12) and was consistent with the Swedish Legislation on Animal Experimentation (Animal Welfare Act SFS1998:56) and the European Communities Council Directive (86/609/EEC).

### The MCSF Test

The MCSF is described in detail elsewhere (Meyerson et al., 2006; Roman and Colombo, 2009). Briefly, the arena (Figure 1B) is 100 × 100 cm and is divided into 10 zones: The center (70 × 70 cm) with a central circle (CTRCI) 25 cm in diameter. From the center are the three corridors accessible, in one corner, there is a covered shelter called the dark corner room (DCR), opposite the DCR is the slightly elevated hurdle with a hole-board for nose pokes. Along the fourth side is the elevated and illuminated wire mesh bridge with entrance, which is accessed from the slope. The arena is illuminated from above and by a bridge lamp to the following light conditions (lux): DCR < 1; CTRCI 15; corridors and hurdle 5–10; bridge 500. The animals were started in the center facing the side not leading to a corridor.

### The EPM Test

The EPM arena (Figure 1C) consists of two open and two closed arms with open endings that together form a cross. Each arm is 40 cm long, 10 cm wide, and covered by a black rubber mat. The walls around the closed arms are black and 40 cm high, and the entire arena is elevated 51 cm from the floor. The central square (CTRSQ, 10 × 10 cm) where the arms meet is considered its own zone. The arena is illuminated from above to a level of 100 lux on the open arms. The animals were started in the CTRSQ facing one of the open arms.

### The OF Test, With and Without Start Box

The OF arena (Figure 1D) is circular, 90 cm in diameter, with a 35-cm high black outer wall and black wire-mesh floor, 10 mm between bars. The arena is divided into three zones by concentric circles: The center (CTROF), 30 cm in diameter; the inner circle (IC), 15 cm wide; and the outer circle (OC), 15 cm wide. The arena is illuminated from above to a level of 100 lux in the center. The start box (25 × 25 × 25 cm) was, when used, attached to the outside of the arena and accessible by a door (8 cm in diameter) in the outer wall. Animals in trials without start box started in the OC facing the wall and when the start box was used, the animals started in the start box.

### The SPB Test

The SPB arena is a square 50 × 50 cm with approximately 2 cm of wood-chip bedding on the floor. The arena is illuminated from above to a level of 10 lux. Prior to testing the animals were habituated to the arena; on two consecutive days each animal spent 5 min exploring the arena alone.

The animals were tested in unfamiliar, weight-matched (<15% body weight difference) pairs and were individually marked on the tip or base of the tail with a xylene-free marker pen to facilitate individual-based behavioral analysis. Directly before testing the animals were isolated for 3.5 h (Niesink and Van Ree, 1989) in type III cages (42.5 × 26.5 × 15 cm). The animals remained in vocal, visual, and olfactory contact with others and had access to food and water. The animals were started directly from their isolation cages into opposite corners of the arena.

### Behavioral Recordings

All test sessions started by running an age-matched out-of-test rat in the arena to avoid any first-in-line effects, where after the experimental rats followed in random order. All tests were recorded from above by video cameras. Recording and observation started immediately after a rat was placed in an arena and lasted for 20 min in all tests except in the SPB test, which lasted 15 min. After each test, the arenas were cleaned with 10% ethanol solution and in the SPB arena the wood-chip bedding was replaced. The operator observed the tests from a room adjacent to the testing room; EthoVision XT (Noldus Inc., Wageningen, Netherlands) was used for manual scoring and automatic tracking. The combination of manual and automatic behavioral recording was determined based on the properties of each behavioral test; lighting conditions, partial and/or complete field of view obstructions, and the type of behaviors recorded. Manual scoring was performed by two trained observers to ensure accurate and reliable identification of the behavioral parameters.

In the MCSF test, manual scoring was used to register latency (L, s) to first visit, frequency (F) of visits and duration (D, s) of time spent in each zone as well as the number of rearing, grooming, and stretched attend postures (SAPs). Nose pokes in the hurdle were recorded by a photocell. Automatic tracking with track smoothing (based on ±10 samples around each sample point) was used to determine mean velocity (cm/s) and total distance (cm) traveled. Track smoothing was used due to the varied conditions in the arena. After each trial, the number of fecal boli and urinations were counted.

For the EPM and the OF tests, automatic tracking was used to determine latency, frequency, and duration in each zone, as well as mean velocity and total distance traveled. The total number of rearing, grooming, and SAPs were manually scored and after each trial the number of fecal boli was counted. Additionally, in the EPM the number of urinations was noted.

For the SPB test, only manual scoring was used. The quality of contact within the pair was scored as no physical contact, contact behavior, or vigorous play (latency, frequency, and duration). The play behavior of the individual animal was quantified by scoring the instances of performing a pounce, pin, or climbing over its play partner (latency, frequency, and duration).

From these descriptive parameters, additional parameters were derived. In the MCSF, EPM, and OF tests, the duration per visit (D/F, s), total activity (sum of all zone frequencies), percental frequency [%F (zone frequency/total activity)], and percental duration [%D (zone duration/test time)] were calculated. For the MCSF, the latency to leave the center for the first time (L leave, s) and the zone measures (F, D, D/F, %F, and %D) for the sum of the corridors (total corridor, TOTCORR) were determined. For the SPB test, the percental duration [%D (behavior duration/test time)] of the contact quality measurements were determined.

### Adult Reference Data

To investigate the influence of age on the performance in the MCSF test, adult male reference data were obtained from Palm et al. (2014) and Momeni and Roman (2014). The reference data were obtained from animals from the same supplier and the animals were housed and tested in the same animal facility and under the same conditions as the present adolescent cohort.

### Statistical Analysis

Classical statistical analysis was carried out in Statistica 13 (TIBCO Software Inc., Tulsa, OK, United States). The parameters were examined for normality with Shapiro–Wilk’s *W*-test, the majority of the parameters were not normally distributed and thus, non-parametric statistics were used. Between-subject differences with more than two groups were examined with Kruskal–Wallis ANOVA by ranks, with *post hoc* Mann–Whitney *U*-test with continuity correction where appropriate. Differences over time within one MCSF trial were examined with the Friedman test with *post hoc* Wilcoxon matched pairs test when significant. Differences between the two trials in the MCSF for the repeatedly tested group were examined with Wilcoxon matched pairs test. Differences between categorical parameters were examined with Pearson Chi-squared test. Correlations were examined with Spearman rank order test. Tests were considered significant at *p* < 0.05.

The trend analysis, a rank-order procedure that group several behavioral parameters in the MCSF into functional behavioral categories, was performed as previously described (Meyerson et al., 2013). In this study only the ranked parameters, not the summed categories, were used.

#### Multivariate Data Analysis

Multivariate statistical analysis was carried out in SIMCA 14 (Umetrics AB, Umeå, Sweden). Three types of multivariate analyses were performed: principal component analysis (PCA) where the relationship between several *X*:s (e.g., behavioral parameters from one test) is examined; partial least squares projections to latent structures (PLS) analysis, examining the relationship between several *X*:s to several *Y*:s (e.g., behavioral parameters from two different tests); and PLS-discriminant analysis (PLS-DA), where the relationship between several *X*:s and one *Y* (e.g., behavioral parameters by a classification) is examined. The autofit option was used in the creation of each model; this generates a model with the maximal number of significant components. Components were excluded if they had eigenvalue <2 or large negative *Q*^2^-values. Latencies, percental frequency, and percental duration were not included in the multivariate statistical analyses. Variables with minimal variance were excluded when advised by the software.

For each analysis, two-dimensional score and loading plots were generated from the first two components. The score plot shows the relationship between the individuals as it summarizes the variables for each individual in each component to a *t*-score. When the components are plotted against each other it enables interpretation of the relatedness between individuals and identification of groups and outliers. The loading plot shows the opposite, i.e., summarizes all individuals for each parameter to a *p*-loading for PCAs and *w* ^∗^ *c*-loadings for both types of PLS analyses and enables grouping of closely related parameters. Graphical interpretation is made by comparing the spatial relationship between two points, if the position of each point is represented as a straight line connecting the point and origo, two points then form an angle which relates to the relationship between them. A narrow angle indicates that the points co-vary in a positive manner in the model, i.e., they have positive correlation between them, a 90° angle indicate that the points are independent and if the points form a straight line through origo they are negatively correlated to each other.

Additional variables derived from the models are, in PLS analysis, the *t*[1]/*u*[1] scores, latent variables describing the first summary of all the *X* variables (*t*[1]), and all the *Y* variables (*u*[1]) and thus the internal relationship between the *X* and the *Y*. Derived from PLS-DA are the coefficients for each variable corresponding to scaled and centered regression coefficients obtained when the PLS-DA model is rewritten as a regression model; the variable importance to the projection (VIP), values inferring how well a variable explains the variance in *X* and its correlation to *Y*; and a predicted *Y*-value, i.e., a model prediction of each individual’s class.

## Results

Two individuals from the MCSF-SPB group were excluded from the analysis due to technical issues during the MCSF trials, which led to incomplete recordings. Their respective SPB partners were not excluded.

The animals weighed between 93.4 and 158.8 g (mean 131.7 g) when the first behavioral test was conducted and between 129.0 and 202.1 g (mean 176.1 g) in the second.

### Adolescent Behavioral Types

**FIGURE 2:**
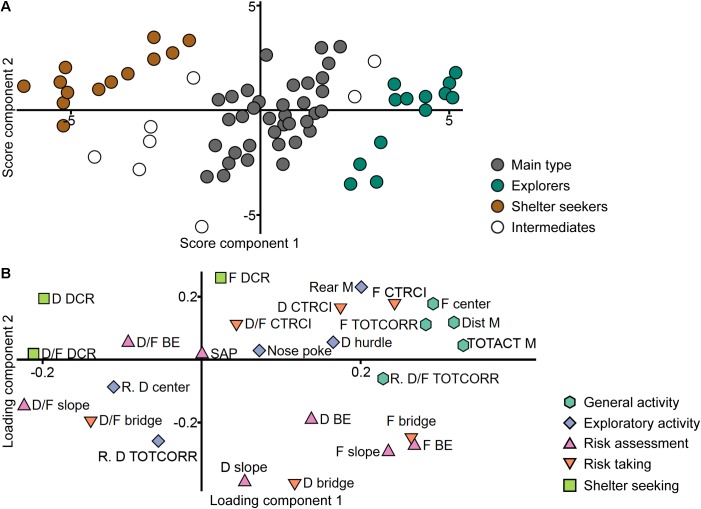
Identification of the behavioral types. Scatter plots of **(A)** individual scores and **(B)** variable loadings from the PCA (*n* = 70, three components, *R*^2^*X* = 0.558, *Q*^2^ = 0.333) of the ranked trend analysis parameters in the MCSF test. Score plot colored according to the clusters used to classify the animals into the behavioral types. Loading plot colored according to the functional behavioral categories established in adult animals (Meyerson et al., 2013). BE, bridge entrance; CTRCI, central circle; D, duration; DCR, dark corner room; Dist, distance; F, frequency; M, MCSF; R., ranking reversed; SAP, stretched attend posture; TOTACT, total activity; TOTCORR, total corridor.

A PCA was made from the ranked parameters of the trend analysis (*n* = 70, three components, *R*^2^*X* = 0.558, *Q*^2^ = 0.333), in an attempt to find the functional behavioral categories previously established for adult animals (Meyerson et al., 2013). In the loading plot (Figure 2B), there is an overlap between slope, bridge entrance, and bridge parameters, especially the frequencies and durations that load in the lower right quadrant and to some extent the duration per visit loading in the lower left. This is in contrast to adults (Meyerson et al., 2013) where these zones are separated and thus included in different risk-associated categories in the trend analysis. Furthermore, parameters from the two activity categories (general and exploratory activity) load in the same direction with large positive contribution of component 1 and low to moderate contribution of component 2 and does not separate as clearly as in adults (Meyerson et al., 2013). In all, the behavioral categories used in the adult trend analysis do not emerge from the adolescent data set. On the contrary, a pattern emerges from the score plot (Figure 2A); here the individuals are clustered in three distinct groups with a few intermediate individuals. These score plot clusters were chosen as identifiers in the creation of a new classification into three groups (62 individuals were included in the classification; 8 intermediate individuals did not receive any classification).

**FIGURE 3:**
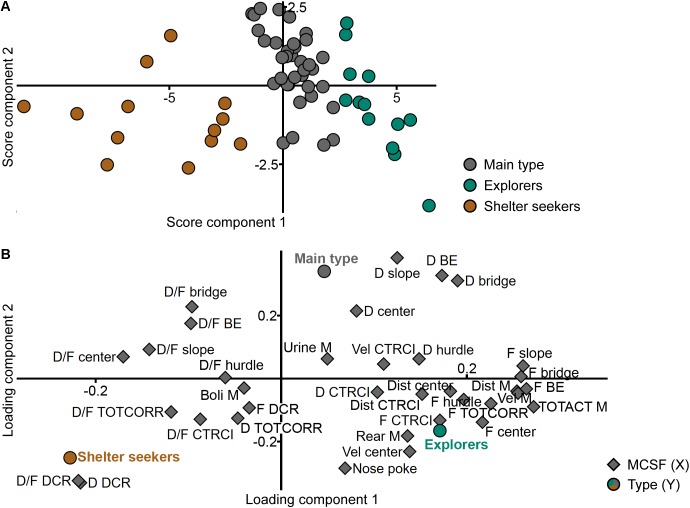
Validation of the behavioral types. Scatter plots of **(A)** individual scores and **(B)** variable loadings from the PLS-DA (*n* = 62, one significant component: *R*^2^*X* = 0.359, *R*^2^*Y* = 0.343, *Q*^2^ = 0.277; a second, non-significant component was added to obtain a two-dimensional model for visualization: *R*^2^*X* = 0.080, *R*^2^*Y* = 0.143, *Q*^2^ = –0.046) of the MCSF parameters (*X*) by behavioral type (*Y*). BE, bridge entrance; CTRCI, central circle; D, duration; DCR, dark corner room; Dist, distance; F, frequency; M, MCSF; TOTACT, total activity; TOTCORR, total corridor; Vel, velocity.

The new classification was validated against the adolescent MCSF dataset in a PLS-DA (*n* = 62, one significant component: *R*^2^*X* = 0.359, *R*^2^*Y* = 0.343, *Q*^2^ = 0.277; a second, non-significant component was added to obtain a two-dimensional model for visualization: *R*^2^*X* = 0.080, *R*^2^*Y* = 0.143, *Q*^2^ = -0.046). The distinction between the groups is seen in the score plot (Figure 3A) and parameters important for the distinction were identified in the loading and regression coefficient plots (Figure 3B and Supplementary Figure 1, respectively). The group loading with high positive contribution of the first component in the score plot (Figure 3A) was named Explorers (*n* = 13) due to its correlation to high velocity and long distance in the arena, high numbers of rearings and visits to the center and, in general, short durations per visit to the individual zones (Figure 3B and Supplementary Figure 1A). The opposite group, with negative contribution of the first component in the score plot (Figure 3A), was named Shelter seekers (*n* = 13). This group correlates to long duration and duration per visit in the DCR and with short durations in the bridge, bridge entrance and center zones, and low distance, velocity, and total activity in the arena (Figure 3B and Supplementary Figure 1B). The remaining group was named Main type (*n* = 36) due to the large number of animals in the classification and the lack of highly distinguishable correlated variables. This type mainly differs from the other two in the second, non-significant component while loading in between Explorers and Shelter seekers in the significant first component, as seen in Figure 3A and in Figure 3B by the distribution of the type (*Y*) variables. Hence, few variables were significant among the regression coefficients (Supplementary Figure 1C) but in general the Main type classification correlated with long durations in the slope, bridge entrance, bridge and center zones, and with short duration and duration per visit in the DCR (Figure 3B and Supplementary Figure 1C); parameters important for the second component as indicated by the VIP score (Supplementary Figure 2). The behavioral type classification is summarized in Table 1.

**Table 1:** Summary of the division of the adolescent animals into behavioral types.

	Main type	Explorers	Shelter seekers
Number of individuals	36	13	13
Characteristics	↔ Activity	↑ Activity	↓ Risk and activity
	(↑) Risk		↑ Shelter seeking
	(↓) Shelter seeking		


### The MCSF

**FIGURE 4:**
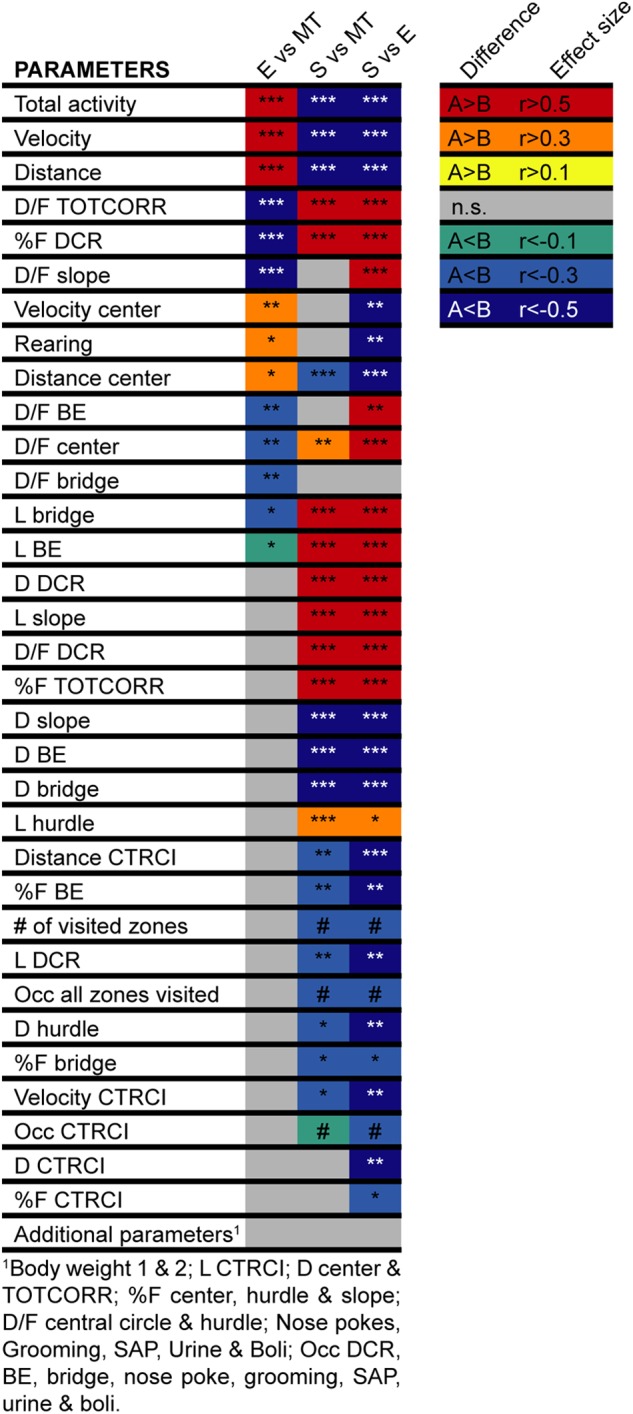
Heatmap of the differences between the behavioral types in the MCSF. Heatmap of the difference in MCSF parameters between rats classified as Explorers (E, *n* = 13), Shelter seekers (S, *n* = 13), or Main type (MT, *n* = 36). The colored squares indicate the direction (red–yellow hues indicate positive effect size, i.e., that the comparing group rank higher, blue–green hues indicate negative effects sizes, i.e., lower ranking) and the level (darker–lighter within each hue category) of the effects size calculated for the significant differences (Fritz et al., 2012). ^∗^*p* < 0.05, ^∗∗^*p* < 0.01, ^∗∗∗^*p* < 0.001, *post hoc* Mann–Whitney *U*-test after significant Kruskal–Wallis test; #*p* < 0.05 Pearson Chi-square test. BE, bridge entrance; CTRCI, central circle; DCR, dark corner room; D, duration; D/F, duration per visit; E, explorer; F, frequency; L, latency; MT, Main type; n.s., non-significant; Occ, occurrence; S, shelter seeker; SAP, stretched attend posture; TOTCORR, total corridor.

The animals were first tested in the MCSF at five (*n* = 23) or six (*n* = 47) weeks of age and thus had the MCSF as either the first or second behavioral test. There were a few parameters showing differences dependent on order; animals that had the MCSF as their second behavioral test had shorter latency to leave the center directly when the test started and had shorter latencies to two of the corridors and to the hurdle. They also had shorter duration (as seconds and percentage) and duration per visit to the center than the animals that were tested first in the MCSF (data not shown). However, there was no relationship between if the animals had the MCSF as first or second behavioral test and which behavioral type each animal was classified as (Supplementary Table 1A). Additionally, there was no relationship between assigned behavioral type and the corridor initially used to exit the center in the MCSF test or which complementary test each animal had (Supplementary Table 1B,C). Lastly, as seen in Figure 4, animals in the three types did not differ in body weight neither at the time of the first behavioral test nor at the second behavioral test.

**FIGURE 5:**
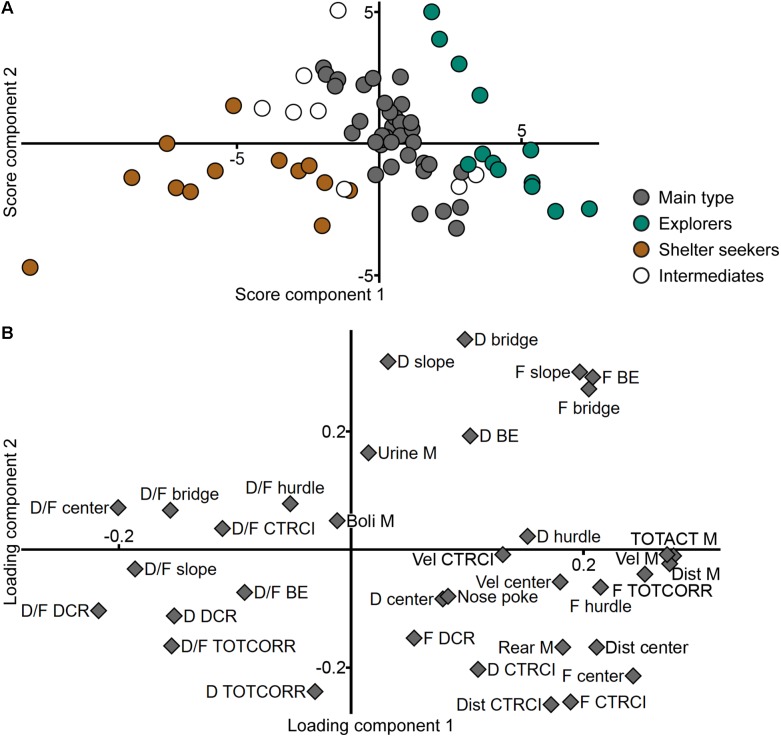
The behavioral types and performance in the MCSF. Scatter plots of **(A)** individual scores and **(B)** variable loadings from the PCA (*n* = 70, four components, *R*^2^*X* = 0.626, *Q*^2^ = 0.286) of the MCSF parameters. The score plot is colored according to behavioral type. BE, bridge entrance; CTRCI, central circle; D, duration; DCR, dark corner room; Dist, distance; F, frequency; M, MCSF; TOTACT, total activity; TOTCORR, total corridor; Vel, velocity.

The result of the PCA of the parameters in the MCSF (*n* = 70, four components, *R*^2^*X* = 0.626, *Q*^2^ = 0.286) can be viewed in Figure 5. In the score plot (Figure 5A), the individuals are spread out across all quadrants but if colored according to behavioral type the grouping is still visible with Main type loading heavily in the upper right quadrant close to origo and fanning out equally into the upper left and lower right quadrants. Of the Explorers, two-thirds load in the lower right quadrant and the rest in the upper right quadrant, but further from origo than the Main type. Shelter seekers load all but one in the lower left quadrant. The animals classified as intermediates show a larger overlap to the types than in the first PCA of the ranked parameters from the trend analysis (Figure 3A). In the loading plot (Figure 5B), activity parameters such as total activity, velocity, distance, and number of visits to the corridors load close together with high positive contribution of component 1 and low contribution of component 2. Close to these parameters but with lower contribution of component 1, load duration and number of visits to the hurdle and velocity in the center and CTRCI. Opposite them, load the duration per visit in most of the individual zones. Duration and duration per visit to the DCR and corridors load together in the lower left quadrant. The duration and number of visits to the slope, bridge entrance, and bridge load together in the upper right quadrant. Spread out in the lower right quadrant are the remaining parameters regarding the center and CTRCI (duration, number of visits, and distance) together with the number of rearings, nose pokes, and visits to the DCR. The number of fecal boli load close to origo and the number of urinations have low contribution of component 1 and modest contribution of component 2.

**FIGURE 6:**
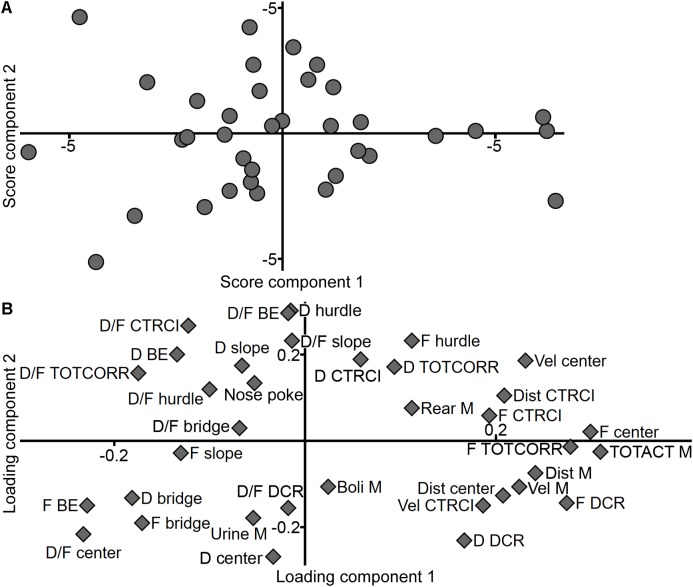
The Main type and performance in the MCSF. Scatter plots of **(A)** individual scores and **(B)** variable loadings from the PCA (*n* = 36, two components, *R*^2^*X* = 0.371, *Q*^2^ = 0.073) of the MCSF parameters among the animals classified as Main type. BE, bridge entrance; CTRCI, central circle; D, duration; DCR, dark corner room; Dist, distance; F, frequency; M, MCSF; TOTACT, total activity; TOTCORR, total corridor; Vel, velocity.

An additional PCA of only the Main type animals (Figure 6; *n* = 36, two components, *R*^2^*X* = 0.371, *Q*^2^ = 0.073) reveal the internal individual variance within this group (Figure 6A) and a different variable loading pattern (Figure 6B) than when the behavioral extremes of Explorers and Shelter seekers are included. In the score plot (Figure 6A), the individuals show a somewhat even distribution across the two-component space. In the loading plot (Figure 6B), the activity parameters are more separated than seen in Figure 5B; total activity remains with high positive contribution of component 1 and low contribution of component 2, together with number of visits to the center and corridors. Distance and velocity load further down in the lower right quadrant, together with distance in the center, velocity in the CTRCI, as well as frequency and duration in the DCR. Most of the other CTRCI parameters (frequency, duration, and distance) load in the upper right quadrant with duration spent in the corridors, velocity in the center, and number of rearings and visits to the hurdle. In the upper left quadrant load most of the duration per visit parameters with number of nose pokes and duration spent in the hurdle, slope, and bridge entrance zones. Frequency to the slope, bridge entrance, and bridge load in the lower left quadrant together with duration in the center and bridge, duration per visit to the center, and DCR and number of urinations. Number of fecal boli load in the lower right quadrant with low contribution of component 1 and moderate contribution of component 2, separate from the other parameters in the quadrant which have much higher contribution of component 1.

**FIGURE 7:**
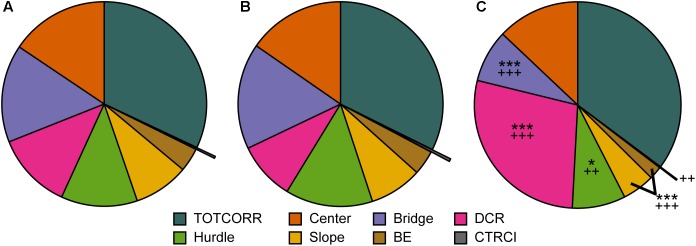
The behavioral types and relative time spent in the zones of the MCSF. Mean percental duration in the zones of the MCSF in **(A)** Main type (*n* = 36), **(B)** Explorers (*n* = 13), and **(C)** Shelter seekers (*n* = 13). ^∗^*p* < 0.05, ^∗∗∗^*p* < 0.001 relative to Main type; ^++^*p* < 0.01, ^+++^*p* < 0.001 relative to Explorers (*post hoc* Mann–Whitney *U*-test). BE, bridge entrance; CTRCI, central circle; DCR, dark corner room; TOTCORR, total corridor.

**FIGURE 8:**
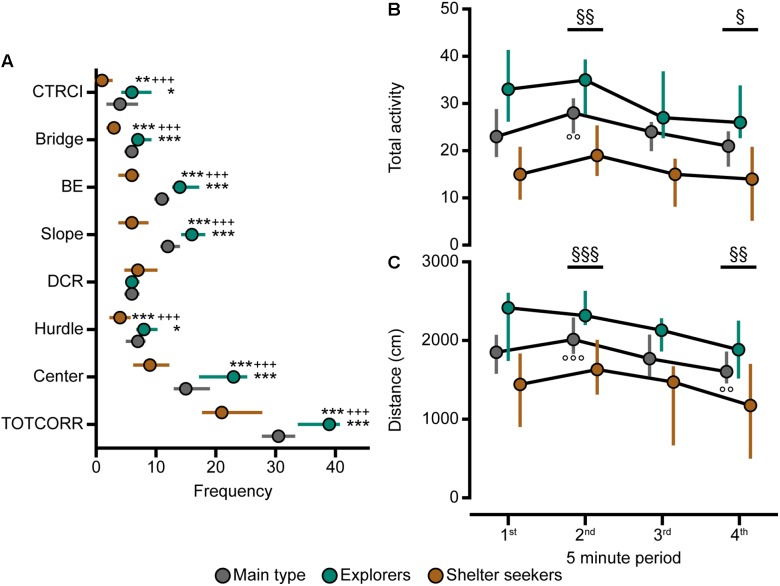
The behavioral types, number of visits to the zones, and activity over time in the MCSF. **(A)** Frequency of visits to the zones in the whole 20-min trial, **(B)** total activity and **(C)** distance traveled during the four 5-min periods of the MCSF test, in Main type (*n* = 36), Explorers (*n* = 13), and Shelter seekers (*n* = 13). Data are presented as median with upper and lower quartiles. ^∗^*p* < 0.05, ^∗∗^*p* < 0.01, ^∗∗∗^*p* < 0.001 relative to Main type; ^+++^*p* < 0.001 relative to Explorers (Mann–Whitney *U*-test). ^§^*p* < 0.05, ^§§^*p* < 0.01, ^§§§^*p* < 0.001 for the whole cohort compared to the first 5 min; ^∘∘^*p* < 0.01, ^∘∘∘^*p* < 0.001 for the Main type compared to the first 5 min (*post hoc* Wilcoxon matched pairs test). All three behavioral types are significantly different from each other in total activity and distance at all four intra-trial time points (*p* < 0.05, additional levels of significance not defined; *post hoc* Mann–Whitney *U*-test). BE, bridge entrance; CTRCI, central circle; DCR, dark corner room; TOTCORR, total corridor.

Classical statistical analysis of the MCSF parameters comparing the three behavioral types confirms the widespread difference among them; 16 out of the 71 parameters exhibit differences between all three behavioral types and an additional 30 parameters have differences between at least two of the types (Figure 4, 7, 8A). As seen in Figure 7, Main types and Explorers spent similar amount of time in the different zones while Shelter seekers spent less time in the bridge, hurdle, slope, and bridge entrance zones compared to both Main types and Explorers. Shelter seekers also spent less time in the CTRCI compared to Explorers and more time in the DCR than both other behavioral types. As further shown in Figure 8A, the behavioral types differed in number of visits to all zones except to the DCR where the frequency was comparable in the different types. In the other zones, Shelter seekers had the lowest frequencies and Explorers the highest. Additionally, as shown in Figure 4, the Explorers had higher scores of the activity parameters total activity, distance, and velocity than both Main types and Shelter seekers, and Main types in turn scored higher in these activity parameters than Shelter seekers.

Analysis of the activity in the MCSF over time revealed that for the whole cohort the total activity and distance traveled increased from the first 5-min period to the second while it decreased when comparing the first to the last 5-min period (Supplementary Table 2, *p*-values in Figure 8B,C). When the cohort was divided into the behavioral types (Figure 8B,C), Shelter seekers and Explorers were stable in activity over time while the Main type displayed an increase in total activity and distance from the first to the second 5-min period, as well as decreased distance traveled between period 1 and 4. Additionally, at all time-points Explorers had higher activity than both Main type and Shelter seekers, and the Main type had higher activity than Shelter seekers.

### Repeated Testing in the MCSF

**FIGURE 9:**
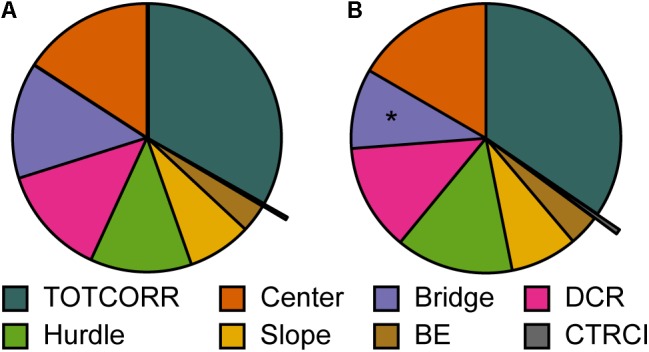
Repeated testing and relative time spent in the zones of the MCSF. Mean percental duration in the zones of the MCSF in **(A)** trial 1 and **(B)** trial 2 (*n* = 12). ^∗^*p* > 0.05 relative to trial 1 (Wilcoxon matched pairs test). BE, bridge entrance; CTRCI, central circle; DCR, dark corner room; TOTCORR, total corridor.

**FIGURE 10:**
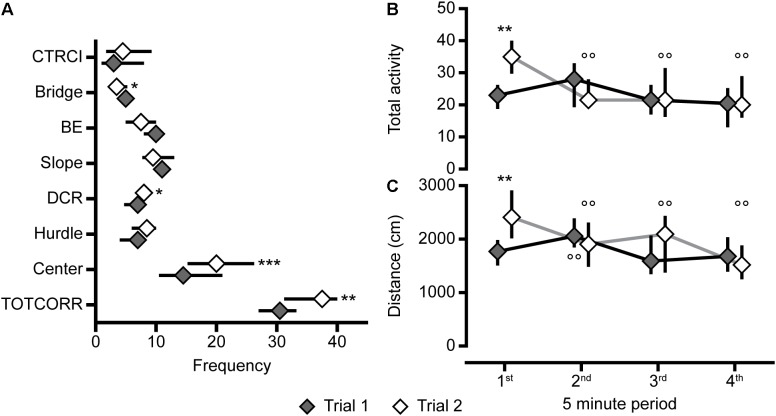
Repeated testing, number of visits to the zones, and activity over time in the MCSF. **(A)** Frequency of visits to the zones in the whole 20-min trials, and **(B)** total activity and **(C)** distance traveled in the four 5-min periods of the MCSF test in trials 1 and 2 of the animals repeatedly tested in the MCSF (*n* = 12). Data are presented as median with upper and lower quartiles. ^∗^*p* < 0.05, ^∗∗^*p* < 0.01, ^∗∗∗^*p* < 0.001 relative to trial 1 (Wilcoxon matched pairs test); ^∘∘^*p* < 0.01, compared to the first 5 min in the respective trial (*post hoc* Wilcoxon matched pairs test). BE, bridge entrance; CTRCI, central circle; DCR, dark corner room; TOTCORR, total corridor.

Twelve animals were tested repeatedly in the MCSF, once at 5 weeks of age and the second time 1 week later. Figure 9 shows the time spent in each individual zone in the first and second trial (Figure 9A,B, respectively). The distribution was similar between the trials, except for the bridge where the animals had shorter duration in the second trial compared to the first. The number of visits to the individual zones (Figure 10A) showed more changes; the center, corridors, and DCR were visited more times in the second trial than in the first, while the bridge received fewer visits. Additionally, Supplementary Table 3 shows that in the second trial the animals left the center faster and had shorter latencies to the individual zones, except to the CTRCI, bridge entrance, and DCR, in which the latencies were similar in the two trials. The total activity was higher in the second trial while other activity parameters (distance, rearing, and velocity) were unchanged. Analysis of the activity over time within each of the trials (Figure 10B,C) revealed that during the first 5 min the animals had higher total activity as well as longer distance traveled in the second trial than in the first. In the second trial, the activity then decreased in the following 5-min periods compared to the first 5 min, which is a different pattern compared to the performance in trial 1, and to the whole study cohort (Supplementary Table 2).

### The MCSF and the Other Exploratory Tests

**FIGURE 11:**
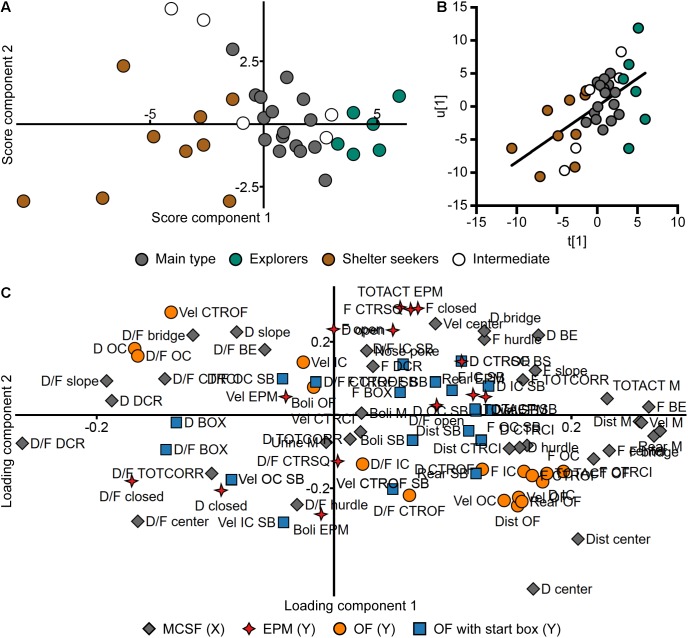
Relationship between the MCSF and the other exploratory tests. Scatter plots of **(A)** individual scores and **(C)** variable loadings from the PLS analysis (*n* = 36, four components, *R*^2^*X* = 0.639, *R*^2^*Y* = 0.520, *Q*^2^ = –0.087) of the parameters from the MCSF (*X*) and EPM, OF, and OF with start box (*Y*). Score plot colored according to behavioral type, loading plot according to test. An enlarged version of **(C)** is available in Supplementary Figure 3. **(B)** Correlation between the latent variables *t*[1] and *u*[1]. The *t*/*u* score reveals a positive correlation (*p* < 0.001, *r* = 0.57, Spearman rank order correlation). BE, bridge entrance; CTRCI, central circle; CTROF, center open field; CTRSQ, central square; D, duration; DCR, dark corner room; Dist, distance; EPM; elevated plus maze; F, frequency; IC, inner circle; M, MCSF; OC, outer circle; OF, open field; SB, start box (test); TOTACT, total activity; TOTCORR, total corridor; Vel, velocity.

Thirty-six of the animals were tested both in the MCSF and another forced exploration test; the EPM, OF, or OF with start box (Supplementary Table 2). A PLS analysis (*n* = 36, four components, *R*^2^*X* = 0.639, *R*^2^*Y* = 0.520, *Q*^2^ = -0.087) was made to investigate the relationship between the MCSF parameters (*X*) and parameters from the other tests (*Y*). In the loading plot (Figure 11C and in an enlarged version in Supplementary Figure 3), the EPM parameters load on a positive diagonal between the upper right and lower left quadrants while OF parameters load on the opposite diagonal between the upper left and lower right quadrants. The MCSF and the OF with start box parameters are spread in all four quadrants. Among the EPM parameters, the duration and duration per visit to the closed arms as well as number of fecal boli load in the lower left quadrant. In the same quadrant load the duration per visit to the hurdle, center, corridors, and DCR of the MCSF, and the duration and duration per visit to the start box in the OF with start box. Most of the OF parameters load in the lower right quadrant close to duration and frequency of the center and CTRCI of the MCSF, and in the same direction as the MCSF activity parameters distance, velocity, rearing, and total activity. The OF parameters that load in the opposite end are the thigmotaxis-associated parameters (duration and duration per visit in the OC) and velocity in the center zone of the OF. In the same quadrant (the upper left) load the MCSF parameters duration spent in the DCR and slope and duration per visit to the slope, CTRCI, bridge, and bridge entrance. In the score plot (Figure 11A) the division into the behavioral types is still visible; the Explorers have the highest contribution of component 1, Shelter seekers have all negative scores in component 1 while the Main type load in between. The latent variables from the PLS analysis (Figure 11B) show a positive correlation, indicating a relationship between *X* and *Y*, i.e., a relationship between the performance in the MCSF and the other behavioral tests with forced exploration.

Based on the loading plot of the PLS analysis (Figure 11C), certain groups of parameters were selected for conventional correlative analysis (Table 2). The MCSF duration per visits that loaded in the lower left quadrant (center, DCR, corridors, and hurdle) were examined for correlations to the parameters from the closed arm of the EPM and the start box of the OF with start box (Table 2A). However, despite the close loadings in the PLS analysis, none of the correlations were significant. EPM activity (total activity, distance, velocity, and rearing) and open arm parameters were examined for their relationship with parameters of the bridge in the MCSF (Table 2B). Despite the similarity in design and interpretation, no correlation between the bridge and open arm parameters was found. On the contrary, the frequency and duration on the bridge in the MCSF showed positive correlations to the total distance traveled and the number of rearings in the EPM. Since many of the OF parameters loaded close to parameters of the center in the MCSF they were examined for correlations (Table 2C). The frequency and duration in the center showed positive correlations to the activity parameters of the OF (total activity, velocity, and number of rearings), duration spent in the center also correlated to the distance traveled in the OF and the distance traveled in the center of the MCSF correlated to the velocity and number of rearings in the OF. Lastly, the consistency of the different activity parameters and number of fecal boli were examined between the MCSF and the different tests (Table 3). The total activity, distance traveled, and number of fecal boli did not correlate between the MCSF and the other tests. However, velocity in the MCSF and the OF showed a positive correlation, as did rearing in the MCSF and OF, and MCSF and EPM.

**Table 2:** Correlation matrixes of parameters selected based on the PLS analysis of MCSF parameters (*X*) and EPM, OF, and OF with start box parameters (*Y*).

	D/F	D/F	D/F	D/F	
(A)	center	DCR	hurdle	TOTCORR	
F box	-0.25	-0.19	-0.51	0.39	
D box	0.45	0.38	0.16	0.16	
D/F box	0.47	0.29	0.55	-0.08	
F closed arms	-0.51	-0.04	-0.10	0.11	
D closed arms	-0.04	0.34	-0.29	0.01	
D/F closed arms	0.52	0.04	0.07	-0.10	

	**F**	**D**	**D/F**		
**(B)**	**bridge**	**bridge**	**bridge**		

F open arms	0.08	0.20	0.32		
D open arms	0.34	0.43	0.18		
D/F open arms	0.47	0.46	-0.15		
Total activity EPM	0.29	0.36	0.14		
Distance EPM	0.63*	0.62*	-0.05		
Velocity EPM	0.01	0.13	0.31		
Rearing EPM	0.88***	0.76**	-0.17		
	**F**	**D**	**D/F**	**Distance**	**Velocity**

**(C)**	**center**	**center**	**center**	**center**	**center**

Total activity OF	0.60*	0.82**	-0.02	0.57	0.01
Distance OF	0.43	0.85***	0.10	0.52	-0.15
Velocity OF	0.59*	0.81**	-0.17	0.65*	0.03
Rearing OF	0.61*	0.80**	-0.18	0.64*	-0.06


*Data are presented as Spearman’s rho (ρ), ^∗^*p* < 0.05, ^∗∗^*p* < 0.01, ^∗∗∗^*p* < 0.001 Spearman rank correlation test. D, duration; D/F, duration per visit; EPM, elevated plus maze; F, frequency; MCSF, multivariate concentric square field; OF, open field; PLS, partial least squares projections to latent structures; TOTCORR, total corridor.*

**Table 3:** Correlation matrix of activity parameters, rearing, and number of fecal boli between the MCSF and the other tests.

MCSF	Total activity	Distance	Velocity	Rearing	Boli
EPM	-0.13	0.22	-0.13	0.82**	0.52
OF	0.55	0.54	0.67*	0.61*	-0.24
OF with start box	0.40	0.36	0.31	0.57	-0.09


*Data are presented as Spearman’s rho (ρ), ^∗^*p* < 0.05, ^∗∗^*p* < 0.01 Spearman rank correlation test. EPM, elevated plus maze; MCSF, multivariate concentric square field; OF, open field.*

### The MCSF and Social Play

The relationship between the MCSF and SPB tests was planned to be analyzed as the MCSF-exploratory tests relationship in the “MCSF and the Other Exploratory Tests”, with a PLS analysis. However, this did not yield any significant components and an attempt to visualize the parameters with a PCA was instead made (Supplementary Figure 4). Autofit yielded a model with one significant component (*n* = 22, *R*^2^*X* = 0.272, *Q*^2^ = 0.092), when forced the second non-significant component showed low model variance and prediction values and an eigenvalue below the defined threshold (*R*^2^*X* = 0.120, *Q*^2^ = -0.096, eigen < 2). Taken together this indicates that the covariance between behavior in the MCSF and behavior in the SPB test is low.

### Importance of Age on MCSF Performance

The importance of age on the performance in the MCSF was examined by comparing the present adolescent cohort with the adult reference data from previously published studies (Momeni and Roman, 2014; Palm et al., 2014). The adult cohort were between 10 and 11 weeks old when tested and weighed between 252.4 and 455.9 g (mean 347.7).

**FIGURE 12:**
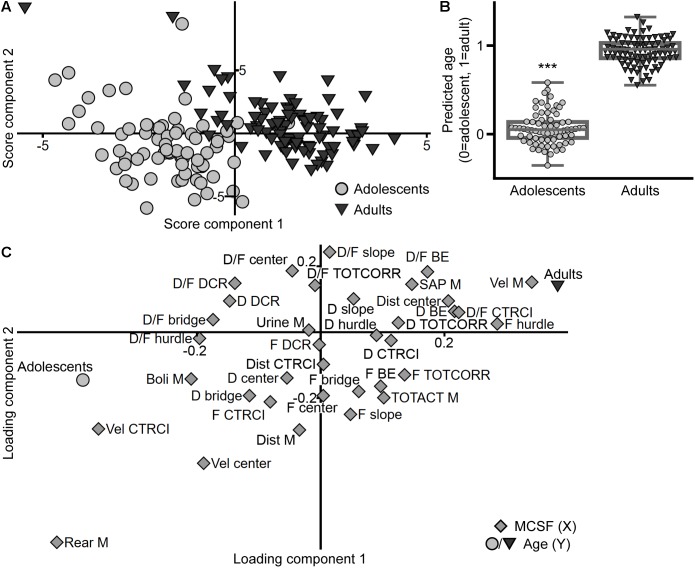
The influence of age on the performance in the MCSF. Scatter plots of **(A)** individual scores and **(C)** variable loadings from the PLS-DA (*n* = 158, four components, *R*^2^*X* = 0.507, *R*^2^*Y* = 0.865, *Q*^2^ = 0.738) of the MCSF parameters (*X*) by age (*Y*). **(B)** Box plot with scatter of the predicted age class (i.e., predicted *Y*-value) derived from the model, data presented as median with upper and lower quartiles, and min and max value. ^∗∗∗^*p* < 0.001 comparing adolescents with adults (Mann–Whitney *U*-test). BE, bridge entrance; CTRCI, central circle; D, duration; DCR, dark corner room; Dist, distance; F, frequency; M, MCSF; SAP, stretched attend posture; TOTACT, total activity; TOTCORR, total corridor; Vel, velocity.

The PLS-DA (*n* = 158, four components, *R*^2^*X* = 0.507, *R*^2^*Y* = 0.865, *Q*^2^ = 0.738) score plot (Figure 12A) shows the separation between the age cohorts. The discrimination between the adolescent and adult animals is further highlighted in the observed versus predicted plot (Figure 12B) where the predicted age class (i.e., the predicted *Y*-value) is significantly different between the two age cohorts. In the loading and regression coefficient plots (Figure 12C and Supplementary Figure 5, respectively), the parameters important for the separation of the age cohorts were identified. Adolescents correlate with long distance traveled in the arena, high number of rearings, high velocity in the center and CTRCI, and high number of fecal boli. On the other hand, adults correlate with high velocity throughout the arena, a high number of SAPs and visits to the hurdle, long duration on the bridge entrance, high duration per visit to the CTRCI, and a long distance traveled in the center.

## Discussion

In the present study, adolescent male rats’ exploratory strategies in the MCSF test were investigated and compared to the performance in the EPM, OF, and SPB tests. The results from the MCSF test revealed three distinct behavioral types; Explorers, Shelter seekers, and Main type. When a subset of the animals was repeatedly tested in the MCSF test, some differences were found relative to the first trial. Moreover, when comparing the performance in the MCSF with that of the reference tests, a relationship between the performance in the MCSF and the other behavioral tests based on forced exploration was found, while no relationship between MCSF performance and social play was found. Finally, adolescent exploratory strategies and behavioral profiles differed from that of adult male rats.

### The MCSF and Adolescent Behavioral Types

As mentioned above, the results from the MCSF test revealed three behavioral types; Explorers, Shelter seekers, and Main types, that were highly distinguishable both using classical statistics (Figure 4, 7–8A) and multivariate data analyses (Figure 3, 5). Explorers were characterized by high activity in the arena and Shelter seekers by their high number of visits to and time spent in the sheltered DCR as well as in the corridors (Figure 3). Previous studies in adult selectively bred alcohol-preferring animals have demonstrated that the corridors attract animals with lower exploratory drive and higher shelter-seeking behavior, and the corridors have therefore been interpreted as semi-sheltered areas (Roman and Colombo, 2009; Roman et al., 2012). Main type animals were located closer to the origo in the analysis defining the types, but when analyzed separately (Figure 6) the internal variation in the Main type group was similar to what have previously been seen in adult rats (Palm et al., 2011; Momeni and Roman, 2014). This indicates that the Explorer and Shelter seeker groups are behaviorally extreme, falling outside of the core behavioral variation.

The difference in behavioral profile between the behavioral types was robust and the classification showed no influence of the tested confounding factors (i.e., body weight, test order, complementary test, or exit corridor in the MCSF). That the MCSF performance was not driven by differences in body weight agrees with previous studies in adult male rats where no (Palm et al., 2011) or only minor (Meyerson et al., 2006) influences of body weight have been found. Test order, however, have in studies using test batteries shown the capability of having considerable effects in adult rats and mice (McIlwain et al., 2001; Paylor et al., 2006; Blokland et al., 2012), although some tests seem to be more sensitive than others. In the present study, there were some differences in MCSF parameters dependent on whether the animal had the MCSF as first or second test but the differences were comparatively few and, as stated above, did not impact the classification into the behavioral types.

Previous studies in adult male rats have used the so-called trend analysis for analysis and interpretation of the behavioral profile in the MCSF (Meyerson et al., 2013). The present analysis of adolescent performance shows that the adult trend analysis is not directly applicable on this age group since the behavioral categories used does not dissociate to the same degree (Figure 2B). Behaviors associated with risk taking and risk assessment in adulthood does not separate in the analysis of adolescent rats, and the same is true for behaviors coded as general and exploratory activity. That the risk taking and risk assessment categories are not distinguishable is probably due to the generally higher levels of risk taking and impulsivity in adolescence (Doremus-Fitzwater et al., 2010; Sturman and Moghaddam, 2011). Correspondingly, higher novelty seeking is probably driving the general and exploratory activity parameters to overlap.

### Repeated Testing in the MCSF

It has previously been demonstrated that adult male rats establish a memory of the arena, that is revealed when repeatedly tested after 1–6 weeks (Meyerson et al., 2006; Roman et al., 2007; Karlsson et al., 2009; Roman and Colombo, 2009; Magara et al., 2015). It can here be concluded that the same is true for adolescent rats as the differences when tested repeatedly were more distinct than when comparing the MCSF performance as the first or second behavioral test, i.e., when first encountering the MCSF at the different ages.

When the animals were repeatedly tested in the MCSF test, the main findings were a lower activity on the bridge, and a higher total activity, driven by more visits to the center and corridors, in the second relative to the first trial (Figure 9, 10A). Activity in risk areas are thought to be driven by a trade-off between potential risks and benefits (Blanchard and Blanchard, 1988; Lima and Dill, 1990). Since the current MCSF set-up assesses basal explorative patterns without possible gains, it is to be expected that risk-taking behavior decreases in the second trial relative to the first. The lower risk-taking behavior on the bridge in the second trial in adolescents herein correspond to that found in most (Meyerson et al., 2006; Roman et al., 2007; Magara et al., 2015), but not all (Roman and Colombo, 2009), studies in adult males. The increase in activity does, however, contrast to the previous studies where adult males consistently decrease their activity in the second trial (Meyerson et al., 2006; Roman et al., 2007; Roman and Colombo, 2009; Magara et al., 2015).

The activity pattern, i.e., total activity and distance moved, over time revealed a modest difference in the two trials (Figure 10B,C). In the second trial, the initial activity was higher than in the first trial where after the activity decreased to the same level as in the first trial. This effect is likely due to an initial defensive response when released in the arena for the first time, manifested as lower initial activity. This is further supported by the longer latency to leave the center for the first time during the first trial compared to the second. An alternative explanation for the initially higher activity in the second trial could be an aversive experience in the first trial resulting in an initial flight response in the second trial, followed by lower activity in the lack of escape routes. However, this seems less likely since the number of rearings and time spent in the different zones, except for the risk area bridge, is similar in both trials, and no increased shelter seeking was observed in the second trial.

### The MCSF and the Reference Tests

Previous studies comparing adult performance in the MCSF test with the EPM and/or OF have found a correspondence between behavioral interpretations from the reference tests to the MCSF. Furthermore, the MCSF captures additional information, which does not have equivalents in the EPM and/or OF tests. Thus, the MCSF has its foundation in the classical behavioral tests but due to its multivariate nature it is able to cover a broader behavioral repertoire (Roman et al., 2007, 2012; Roman and Colombo, 2009). A similar pattern was revealed here in adolescent animals.

The behavioral types identified by use of the MCSF test were distinguishable also when the other forced exploration tests were brought into the analysis, indicating a correspondence between the behavior in the MCSF and the reference tests as the pattern with the behavioral types was preserved (Figure 11A). This is supported by the correlation between the latent variables from the summation of the *X* and *Y* (Figure 11B). Altogether, this indicates an overall relationship between the performance in the MCSF and the other behavioral tests with forced exploration. However, when correlating specific corresponding parameters from the tests the outcome was mixed (Table 3). Velocity in the MCSF and the OF showed a positive correlation, as did rearing in the MCSF and OF, and MCSF and EPM. That rearing, a species-typical behavior, correlated well between the tests is in line with previous studies; in adult rats rearing in the OF and elevated zero maze correlated (Blokland et al., 2012) and in adult mice rearing in the EPM, OF, and LDB showed positive correlations (O’Leary et al., 2013). However, the total activity, distance traveled, and number of fecal boli did not correlate between the MCSF and the other tests. This is in contrast to other studies where locomotion have been found to correlate in the EPM, OF, and LBD tests in adult mice (O’Leary et al., 2013) and adolescent rats (Acevedo et al., 2014). This could be due to methodological differences (e.g., different testing times and/or inter-test intervals) or alternatively, that these behaviors reflect different behavioral qualities in the MCSF compared to the reference tests, due to the varied design of the MCSF arena. For example, to achieve a high total activity in the OF, the animal must enter the risk-associated, inner part of the arena, while MCSF zone transitions can be made between zones of similar quality. It is evident that strategies in a multivariate environment are different from those of more univariate environments (Table 2). For instance, shelter seeking behavior in the DCR was not correlated to shelter seeking in the start box of the OF nor the closed arms of the EPM. Similarly, activity in the risk area bridge was not correlated to activity on the open arms of the EPM.

Altogether, the behavior in the MCSF corresponds to the behavior in the other exploratory tests but not in a linear way, i.e., the same measurement in the different tests does not correlate with each other directly. Additionally, the MCSF contains areas associated with different types of risk-related behaviors, i.e., open and illuminated areas separately and a larger opportunity for risk assessment, and possibilities for shelter seeking. This multivariate design therefore gives a broader behavioral profile but also highlights the importance of the established practice of basing behavioral interpretations on several parameters, as done using the adult trend analysis (Roman et al., 2007, 2012; Roman and Colombo, 2009; Magara et al., 2015) and herein in the identification of the adolescent behavioral types.

In contrast to the relationship between the MCSF and the other forced exploration tests, the covariance between behavior in the MCSF and behavior in the SPB test was low. This is, however, not entirely surprising as environmental exploration and social behaviors are often described as opposing entities, and in adolescent rats a novel environment even inhibits the expression of social play (Vanderschuren et al., 1995). Additionally, social play is very reciprocal and the performance in the SPB test is thus a product depending equally on both play partners whereas the MCSF performance is driven by the internal motivation to explore the environment of each individual. To balance these factors and successfully detangle any relationship between exploration and social behaviors is a methodological challenge, but the present study can at least exclude any overt relationship between exploration in a novel, multivariate environment, and SPB in adolescence.

### Importance of Age on MCSF Performance

The importance of age on the performance in the MCSF was examined by comparing the adolescent cohort with adult reference data from previously published studies using male Wistar rats from the same vendor (Momeni and Roman, 2014; Palm et al., 2014).

A clear separation between adolescent and adult behavioral profiles was revealed in the analysis, further supported by a significant difference in the predicted age class from the model (Figure 12). Notably, risk assessment was less pronounced in adolescents relative to adults, which resembles features of human adolescence (Doremus-Fitzwater et al., 2010; Sturman and Moghaddam, 2011). This is corroborating the finding that parameters related to risk assessment and risk taking does not separate in the analysis of the adolescent cohort alone (Figure 2). Adolescents were characterized by high total distance moved, high number of rearings and fecal boli, and high velocity in open areas (the center and CTRCI), while adults had higher general velocity in the arena, more activity in open areas (long distance traveled in the center and long duration per visit to the CTRCI), and typical risk assessment behaviors, i.e., high number of SAPs and long duration on the bridge entrance (Figure 12C).

Considering the multivariate nature of the MCSF test, several physiological functions, including locomotion, exploration, decision making, defensive behaviors, and cognitive functions, are required to traverse the test. Based on ontological studies, many of these functions have reached the level of adult rats by the time of testing in the present study, but not all. The finding of higher number of fecal boli in adolescents relative to adults agrees with previous studies showing that defecation levels progressively decrease with age in male rats (Masur et al., 1980). Furthermore, it has been demonstrated that exploratory behavior is developing throughout adolescence as activity in the central parts of the OF and on the open arms of the EPM has been shown to increase with age within adolescence (Lynn and Brown, 2009), which can support the finding herein of higher velocity in open areas (interpreted as avoiding the open areas) in adolescent relative to adult rats and higher activity in the open areas (CTRCI) characterizing adult rats. Finally, the number of SAPs in the EPM has previously been shown to increase with age (Almeida et al., 1994), which is in agreement with the findings herein of higher number of SAPs in adult relative to adolescent rats. Thus, one could speculate that with a limited possibility to assess risk, adolescents may benefit from the strategy to move fast in open areas associated with risk for predation, whereas adults can assess the risk and be more active in open areas when the coast is clear.

## General Discussion

The MCSF has since its conception over a decade ago, proven useful in a number of studies using adult rats (Karlsson et al., 2009; Roman et al., 2012; Momeni and Roman, 2014; Palm et al., 2014; Magara et al., 2015) and mice (Augustsson and Meyerson, 2004; Ekmark-Lewén et al., 2010; Ekmark-Lewén et al., 2018). More recently, studies examining adolescent rats (Palm et al., 2013; Berardo et al., 2016; Wille-Bille et al., 2017, 2018) and mice (Stringer et al., 2017) have started using the MCSF as well. In addition, a corresponding test is being developed for adult zebrafish (Roman et al., 2016, 2018). This broad applicability of the MCSF demonstrates the strength of the multivariate approach in assessment of behavior across ages and species.

The current study only included adolescent males, which is a limitation since the importance of including female rats has recently been emphasized (Beery and Zucker, 2011; Becker et al., 2016). Few studies using the MCSF have included adult female rats (Meyerson et al., 2006; Daoura et al., 2010; Lundberg et al., 2017), and only one have performed a direct comparison between males and females. In adult Sprague-Dawley rats, no separation in a PCA was revealed between males and females (Meyerson et al., 2006). When adolescent rats have been assessed using the MCSF, either only one sex was investigated (Palm et al., 2013; Berardo et al., 2016; Wille-Bille et al., 2017) or the effect of sex was not reported (Wille-Bille et al., 2018). Future studies will therefore elucidate potential sex differences in the MCSF performance in adolescent rats.

Behavioral neuroscience is often given as an example of a research area suffering from poor validity and reproducibility. In part, this may be due to insufficient knowledge in behavioral theories resulting in important factors not being considered (Schellinck et al., 2010; Garner et al., 2017). For instance, tests that originally were developed and used for adult rats have been adapted for mice without acknowledging species-specific differences in behavior. Moreover, tests developed and used for adult animals are used to assess adolescent animals without careful evaluation of how to interpret results for adolescents. The present study tries to fill this gap in knowledge regarding age in the MCSF test and aid in further studies of adolescent behavior.

This study was conducted to evaluate explorative strategies in adolescent animals in the MCSF test and to compare the behavior with other commonly used behavioral tests. It can be concluded that in adolescent rats, three distinct behavioral types, i.e., Explorers, Shelter seekers, and Main type, can be found, and adolescent performance differs considerably from that of adults, with low levels of risk assessment behavior characterizing adolescent rats.

## Data Availability

All datasets generated for this study are included in the manuscript and/or the Supplementary Files (Supplementary Data Sheet 1).

## Author Contributions

ER contributed to conception and design of the study, and provided the funding. SL and CH performed the experiments. SL performed the statistical analysis and wrote the first draft of the manuscript. All authors contributed to manuscript revision, read, and approved the submitted version.

## Conflict of Interest Statement

The authors declare that the research was conducted in the absence of any commercial or financial relationships that could be construed as a potential conflict of interest.
