# Supplementary material for: Adolescent Exploratory Strategies and Behavioral Types in the Multivariate Concentric Square FieldTM Test
Source: Front Behav Neurosci. 2019 Mar 4;13:41. doi: 10.3389/fnbeh.2019.00041 (PMC6409336; doi:10.3389/fnbeh.2019.00041)
Supplement: Supplementary file 1 [file Table_1.DOCX]

**Supplementary Table 1.** Cross-tabulation of behavioral type versus (**A**) order of the MCSF test, (**B**) which corridor the animal first left the Center through and (**C**) complementary test (p>0.05, Pearson Chi^2^ test).

| **A.** | Main type | Explorer | Shelter seeker |  |
| --- | --- | --- | --- | --- |
| MCSF first | 13 | 5 | 3 | 21 |
| MCSF second | 23 | 8 | 10 | 41 |
|  | 36 | 13 | 13 | 62 |
| **B.** |  |  |  |  |
| Corridor A | 13 | 5 | 10 | 28 |
| Corridor B | 4 | 2 | 1 | 7 |
| Corridor C | 19 | 6 | 2 | 27 |
|  | 36 | 13 | 13 | 62 |
| **C.** |  |  |  |  |
| Repeated MCSF | 8 | 1 | 2 | 11 |
| EPM | 8 | 0 | 3 | 11 |
| OF | 5 | 3 | 1 | 9 |
| OF with start box | 3 | 3 | 5 | 11 |
| SPB | 12 | 6 | 2 | 20 |
|  | 36 | 13 | 13 | 62 |
