# Supplementary material for: Adolescent Exploratory Strategies and Behavioral Types in the Multivariate Concentric Square FieldTM Test
Source: Front Behav Neurosci. 2019 Mar 4;13:41. doi: 10.3389/fnbeh.2019.00041 (PMC6409336; doi:10.3389/fnbeh.2019.00041)
Supplement: Supplementary file 2 [file Table_2.docx]

**Supplementary Table 2.** Whole cohort results in the behavioral tests (multivariate concentric square field (MCSF), elevated plus maze (EPM), open field (OF), open field with start box (SB) and social play behavior).

| **PARAMETERS** | | Median | Quartiles | | |
| --- | --- | --- | --- | --- | --- |
| **Multivariate concentric square field,** n=70 | | | | | |
|  | L leave | 28.0 | 19.2 | - | 46.6 |
|  | F center | 14.0 | 11.0 | - | 20.0 |
|  | D center | 175.3 | 144.7 | - | 204.4 |
|  | D/F center | 11.8 | 8.3 | - | 14.9 |
|  | Distance center | 1822.9 | 1546.4 | - | 2188.0 |
|  | Velocity center | 10.8 | 9.3 | - | 12.0 |
|  | %F center | 16.6 | 14.9 | - | 19.3 |
|  | %D center | 14.6 | 12.1 | - | 17.0 |
|  | L central circle | 199.9 | 57.8 | - | 335.2 |
|  | F central circle | 3.5 | 1.0 | - | 6.0 |
|  | D central circle | 3.0 | 1.1 | - | 6.2 |
|  | D/F central circle | 0.8 | 0.6 | - | 1.2 |
|  | Distance central circle | 103.1 | 47.1 | - | 165.6 |
|  | Velocity central circle | 29.0 | 18.9 | - | 40.2 |
|  | %F central circle | 3.9 | 1.8 | - | 6.9 |
|  | %D central circle | 0.3 | 0.1 | - | 0.5 |
|  | F total corridor | 30.5 | 27.0 | - | 35.0 |
|  | D total corridor | 382.2 | 351.4 | - | 429.8 |
|  | D/F total corridor | 12.3 | 10.6 | - | 14.6 |
|  | %F total corridor | 33.8 | 31.3 | - | 36.3 |
|  | %D total corridor | 32.0 | 29.4 | - | 35.9 |
|  | L DCR | 133.0 | 46.2 | - | 243.4 |
|  | F DCR | 6.0 | 5.0 | - | 8.0 |
|  | D DCR | 147.4 | 105.2 | - | 219.9 |
|  | D/F DCR | 21.8 | 16.6 | - | 33.7 |
|  | %F DCR | 7.0 | 5.5 | - | 8.8 |
|  | %D DCR | 12.3 | 8.8 | - | 18.3 |
|  | L hurdle | 153.7 | 70.8 | - | 256.3 |
|  | F hurdle | 6.0 | 5.0 | - | 8.0 |
|  | D hurdle | 139.6 | 111.3 | - | 159.1 |
|  | D/F hurdle | 20.0 | 16.6 | - | 24.8 |
|  | %F hurdle | 7.0 | 6.0 | - | 8.3 |
|  | %D hurdle | 11.6 | 9.3 | - | 13.3 |
|  | L slope | 120.8 | 70.4 | - | 250.8 |
|  | F slope | 12.0 | 10.0 | - | 14.0 |
|  | D slope | 90.6 | 74.8 | - | 112.4 |
|  | D/F slope | 8.0 | 6.4 | - | 9.7 |
|  | %F slope | 13.1 | 11.3 | - | 15.0 |
|  | %D slope | 7.6 | 6.2 | - | 9.4 |
|  | L bridge entrance | 176.2 | 112.6 | - | 278.2 |
|  | F bridge entrance | 11.0 | 8.0 | - | 13.0 |
|  | D bridge entrance | 40.8 | 32.3 | - | 50.2 |
|  | D/F bridge entrance | 3.9 | 3.3 | - | 5.0 |
|  | %F bridge entrance | 11.2 | 9.6 | - | 13.2 |
|  | %D bridge entrance | 3.4 | 2.7 | - | 4.2 |
|  | L bridge | 177.3 | 120.4 | - | 284.5 |
|  | F bridge | 5.0 | 4.0 | - | 6.0 |
|  | D bridge | 161.7 | 126.0 | - | 214.0 |
|  | D/F bridge | 32.3 | 27.4 | - | 40.5 |
|  | %F bridge | 5.8 | 4.5 | - | 6.7 |
|  | %D bridge | 13.5 | 10.5 | - | 17.8 |
|  | Total activity MCSF | 91.0 | 79.0 | - | 106.0 |
|  | Distance MCSF | 7109.8 | 6418.1 | - | 7940.7 |
|  | Velocity MCSF | 6.0 | 5.4 | - | 6.7 |
|  | Rearing MCSF | 93.5 | 74.0 | - | 112.0 |
|  | Nose pokes | 1.0 | 0.0 | - | 2.0 |
|  | Grooming MCSF | 1.0 | 0.0 | - | 2.0 |
|  | SAP MCSF | 0.0 | 0.0 | - | 0.0 |
|  | Urine MCSF | 0.0 | 0.0 | - | 1.0 |
|  | Boli MCSF | 0.0 | 0.0 | - | 1.0 |
|  | 1^st^ 5 min total activity | 23.6 | 43.0 | - | 17.0 |
|  | 2^nd^ 5 min total activity | 27.2 | 40.0 | - | 23.0 |
|  | 3^rd^ 5 min total activity | 22.6 | 42.0 | - | 19.0 |
|  | 4^th^ 5 min total activity | 20.6 | 37.0 | - | 17.0 |
|  | 1^st^ 5 min distance | 1800.7 | 2727.9 | - | 1567.9 |
|  | 2^nd^ 5 min distance | 2006.7 | 2795.1 | - | 1824.0 |
|  | 3^rd^ 5 min distance | 1685.4 | 2599.4 | - | 1472.9 |
|  | 4^th^ 5 min distance | 1553.2 | 2583.7 | - | 1398.5 |
| **Elevated plus maze,** n=12 | | | | | |
|  | L open arm | 33.2 | 7.7 | - | 160.4 |
|  | F open arms | 25.5 | 11.5 | - | 40.5 |
|  | D open arms | 38.1 | 19.6 | - | 50.4 |
|  | D/F open arms | 1.5 | 0.9 | - | 2.3 |
|  | %F open arms | 19.3 | 14.2 | - | 22.3 |
|  | %D open arms | 3.2 | 1.6 | - | 4.2 |
|  | L closed arm | 3.7 | 1.4 | - | 8.8 |
|  | F closed arms | 51.0 | 28.0 | - | 72.5 |
|  | D closed arms | 1130.8 | 1055.9 | - | 1149.1 |
|  | D/F closed arms | 21.7 | 14.6 | - | 42.5 |
|  | %F closed arms | 35.4 | 28.9 | - | 39.2 |
|  | %D closed arms | 94.2 | 88.0 | - | 95.8 |
|  | F central square | 67.5 | 40.0 | - | 97.5 |
|  | D central square | 43.0 | 20.6 | - | 84.3 |
|  | D/F central square | 0.8 | 0.4 | - | 1.1 |
|  | %F central square | 47.3 | 46.6 | - | 49.1 |
|  | %D central square | 3.6 | 1.7 | - | 7.0 |
|  | Total activity EPM | 139.0 | 83.5 | - | 202.0 |
|  | Distance EPM | 3225.7 | 2306.6 | - | 3690.7 |
|  | Velocity EPM | 9.1 | 8.8 | - | 9.9 |
|  | Rearing EPM | 22.5 | 16.0 | - | 26.5 |
|  | Grooming EPM | 6.0 | 3.5 | - | 8.0 |
|  | SAP EPM | 2.0 | 0.5 | - | 2.5 |
|  | Urine EPM | 0.0 | 0.0 | - | 0.0 |
|  | Boli EPM | 4.0 | 0.5 | - | 5.0 |
| **Open field,** n=12 | | | | | |
|  | F outer circle | 51.0 | 35.0 | - | 64.0 |
|  | D outer circle | 1125.8 | 1086.7 | - | 1155.7 |
|  | D/F outer circle | 22.2 | 17.1 | - | 33.4 |
|  | %F outer circle | 47.6 | 41.5 | - | 51.7 |
|  | %D outer circle | 93.8 | 90.6 | - | 96.3 |
|  | Velocity outer circle | 9.0 | 8.3 | - | 10.1 |
|  | L inner circle | 25.4 | 11.7 | - | 51.1 |
|  | F inner circle | 47.5 | 28.0 | - | 64.0 |
|  | D inner circle | 58.5 | 32.8 | - | 95.6 |
|  | D/F inner circle | 1.3 | 1.1 | - | 1.8 |
|  | %F inner circle | 43.9 | 41.2 | - | 47.6 |
|  | %D inner circle | 4.9 | 2.7 | - | 8.0 |
|  | Velocity inner circle | 16.0 | 14.0 | - | 18.6 |
|  | L center OF | 115.9 | 55.0 | - | 159.0 |
|  | F center OF | 11.5 | 6.5 | - | 15.0 |
|  | D center OF | 11.0 | 4.9 | - | 19.9 |
|  | D/F center OF | 1.2 | 0.7 | - | 1.3 |
|  | %F center OF | 10.3 | 7.8 | - | 11.5 |
|  | %D center OF | 0.9 | 0.4 | - | 1.7 |
|  | Velocity center OF | 20.2 | 17.3 | - | 24.2 |
|  | Total activity OF | 112.0 | 75.5 | - | 136.5 |
|  | Distance OF | 6389.3 | 5379.3 | - | 7393.2 |
|  | Velocity OF | 9.4 | 8.7 | - | 10.5 |
|  | Rearing OF | 48.0 | 27.5 | - | 67.0 |
|  | Grooming OF | 6.5 | 4.0 | - | 10.5 |
|  | SAP OF | 0.0 | 0.0 | - | 0.0 |
|  | Boli OF | 6.0 | 4.5 | - | 7.0 |
| **Open field with start box**, n=12 | | |  |  |  |
|  | L outer circle SB | 73.6 | 16.3 | - | 100.9 |
|  | F outer circle SB | 62.0 | 37.5 | - | 67.5 |
|  | D outer circle SB | 501.7 | 380.1 | - | 578.4 |
|  | D/F outer circle SB | 9.1 | 7.7 | - | 10.8 |
|  | %F outer circle SB | 48.3 | 41.9 | - | 52.8 |
|  | %D outer circle SB | 40.3 | 30.5 | - | 46.5 |
|  | Velocity outer circle SB | 9.5 | 8.7 | - | 10.0 |
|  | L inner circle SB | 176.9 | 147.5 | - | 219.5 |
|  | F inner circle SB | 33.0 | 23.5 | - | 56.0 |
|  | D inner circle SB | 32.8 | 20.4 | - | 55.4 |
|  | D/F inner circle SB | 0.9 | 0.7 | - | 1.2 |
|  | %F inner circle SB | 31.6 | 23.9 | - | 37.6 |
|  | %D inner circle SB | 2.6 | 1.6 | - | 4.4 |
|  | Velocity inner circle SB | 17.2 | 15.6 | - | 23.0 |
|  | L center SB | 341.9 | 185.3 | - | 584.7 |
|  | F center SB | 11.0 | 7.0 | - | 18.5 |
|  | D center SB | 12.3 | 5.2 | - | 16.5 |
|  | D/F center SB | 0.9 | 0.5 | - | 1.3 |
|  | %F center SB | 9.1 | 7.7 | - | 12.6 |
|  | %D center SB | 1.0 | 0.4 | - | 1.3 |
|  | Velocity center SB | 15.8 | 12.7 | - | 26.0 |
|  | L box return | 106.5 | 69.0 | - | 172.0 |
|  | F box | 13.0 | 11.5 | - | 16.0 |
|  | D box | 672.9 | 584.4 | - | 804.1 |
|  | D/F box | 50.4 | 33.2 | - | 69.3 |
|  | %F box | 11.7 | 9.9 | - | 14.8 |
|  | %D box | 54.1 | 47.0 | - | 64.6 |
|  | Total activity SB | 121.5 | 82.5 | - | 158.5 |
|  | Distance SB | 5530.2 | 4449.2 | - | 6668.3 |
|  | Velocity SB | 7.5 | 6.0 | - | 8.6 |
|  | Rearing SB | 46.0 | 29.0 | - | 80.5 |
|  | Grooming SB | 1.0 | 0.0 | - | 2.0 |
|  | SAP SB | 0.0 | 0.0 | - | 1.0 |
|  | Boli SB | 4.0 | 2.0 | - | 6.0 |
| **Social play behavior**, n=22 | | | | | |
|  | F no contact | 48.5 | 40.0 | - | 58.0 |
|  | D no contact | 570.5 | 353.7 | - | 596.4 |
|  | %D no contact | 59.4 | 36.8 | - | 62.1 |
|  | L social contact | 21.7 | 20.2 | - | 23.4 |
|  | F social contact | 52.0 | 41.0 | - | 67.0 |
|  | D social contact | 389.5 | 314.2 | - | 502.1 |
|  | %D social contact | 40.6 | 32.7 | - | 52.3 |
|  | L play | 179.0 | 88.9 | - | 393.8 |
|  | F play | 2.5 | 0.0 | - | 10.0 |
|  | D play | 31.0 | 0.0 | - | 67.5 |
|  | %D play | 3.2 | 0.0 | - | 7.0 |
|  | L pounce | 153.5 | 90.3 | - | 217.0 |
|  | F pounce | 6.5 | 4.0 | - | 10.0 |
|  | D pounce | 6.3 | 3.2 | - | 9.6 |
|  | L pin | 207.0 | 147.8 | - | 223.8 |
|  | F pin | 0.0 | 0.0 | - | 1.0 |
|  | D pin | 0.0 | 0.0 | - | 0.6 |
|  | L over | 45.4 | 27.6 | - | 63.5 |
|  | F over | 16.5 | 13.0 | - | 22.0 |
|  | D over | 21.2 | 10.7 | - | 27.4 |
| Abbreviations: D, duration (s); DCR, dark corner room; D/F, duration per visit (s); F, frequency; L, latency (s); SAP, stretched attend posture. | | | | | |
