# Supplementary material for: Adolescent Exploratory Strategies and Behavioral Types in the Multivariate Concentric Square FieldTM Test
Source: Front Behav Neurosci. 2019 Mar 4;13:41. doi: 10.3389/fnbeh.2019.00041 (PMC6409336; doi:10.3389/fnbeh.2019.00041)
Supplement: Supplementary file 3 [file Table_3.DOCX]

**Supplementary Table 3.** Results from repeated testing in the multivariate concentric square field™ (MCSF) test in 12 rats.

| **PARAMETERS** | **First trial** | | | | **Second trial** | | | |  |
| --- | --- | --- | --- | --- | --- | --- | --- | --- | --- |
|  | Median | Quartiles | | | Median | Quartiles | | | P value |
| L leave | 40.2 | 16.9 | - | 51.2 | 6.1 | 4.6 | - | 6.9 | ** |
| F center | 14.5 | 11.5 | - | 20.5 | 20.0 | 16.0 | - | 25.0 | ** |
| D center | 202.9 | 172.5 | - | 209.4 | 193.6 | 176.9 | - | 217.0 |  |
| D/F center | 14.1 | 10.2 | - | 15.5 | 10.0 | 8.6 | - | 11.4 | * |
| Distance center | 2221.1 | 1673.7 | - | 2316.9 | 2325.2 | 2247.0 | - | 2776.8 | ** |
| Velocity center | 10.8 | 9.0 | - | 11.5 | 12.7 | 10.8 | - | 14.4 | ** |
| %F center | 18.1 | 15.2 | - | 20.0 | 20.7 | 18.0 | - | 23.1 | ** |
| %D center | 16.9 | 14.5 | - | 17.5 | 16.1 | 14.7 | - | 18.1 |  |
| L central circle | 85.3 | 22.4 | - | 248.2 | 82.6 | 18.8 | - | 449.3 |  |
| F central circle | 3.0 | 1.5 | - | 7.5 | 4.5 | 2.0 | - | 8.0 |  |
| D central circle | 3.4 | 2.8 | - | 6.4 | 3.1 | 1.2 | - | 15.2 |  |
| D/F central circle | 0.9 | 0.7 | - | 2.0 | 0.8 | 0.6 | - | 1.9 |  |
| Distance central circle | 73.4 | 43.4 | - | 183.2 | 107.3 | 54.0 | - | 257.5 |  |
| Velocity central circle | 20.2 | 11.3 | - | 30.5 | 25.2 | 13.8 | - | 34.8 |  |
| %F central circle | 3.4 | 2.2 | - | 7.1 | 4.7 | 2.5 | - | 7.8 |  |
| %D central circle | 0.3 | 0.2 | - | 0.5 | 0.3 | 0.1 | - | 1.3 |  |
| Occ central circle | 12/12 |  |  |  | 11/12 |  |  |  |  |
| F total corridor | 30.5 | 27.5 | - | 33.0 | 37.5 | 32.0 | - | 39.5 | ** |
| D total corridor | 410.7 | 357.6 | - | 431.2 | 399.0 | 349.9 | - | 457.7 |  |
| D/F total corridor | 13.0 | 12.0 | - | 14.6 | 11.3 | 9.0 | - | 13.8 | * |
| %F total corridor | 34.1 | 32.8 | - | 35.5 | 37.6 | 34.6 | - | 39.6 | * |
| %D total corridor | 34.3 | 29.9 | - | 35.9 | 33.3 | 29.2 | - | 38.1 |  |
| L DCR | 74.1 | 43.0 | - | 253.2 | 9.5 | 7.7 | - | 46.3 |  |
| F DCR | 7.0 | 5.0 | - | 7.5 | 8.0 | 7.0 | - | 9.0 | * |
| D DCR | 135.7 | 109.2 | - | 176.3 | 144.9 | 113.0 | - | 192.6 |  |
| D/F DCR | 21.7 | 17.7 | - | 22.8 | 18.2 | 14.5 | - | 23.1 |  |
| %F DCR | 7.3 | 6.1 | - | 7.9 | 7.5 | 7.1 | - | 9.5 |  |
| %D DCR | 11.3 | 9.1 | - | 14.8 | 12.1 | 9.4 | - | 16.1 |  |
| L hurdle | 164.8 | 68.4 | - | 300.1 | 59.5 | 34.1 | - | 134.4 | * |
| F hurdle | 7.0 | 4.5 | - | 8.0 | 8.5 | 6.5 | - | 9.5 |  |
| D hurdle | 147.0 | 131.6 | - | 164.0 | 162.1 | 142.7 | - | 186.2 |  |
| D/F hurdle | 21.6 | 15.7 | - | 30.5 | 20.3 | 17.6 | - | 26.0 |  |
| %F hurdle | 7.9 | 5.7 | - | 9.0 | 8.0 | 6.4 | - | 9.5 |  |
| %D hurdle | 12.3 | 11.0 | - | 13.7 | 13.5 | 11.9 | - | 15.5 |  |
| L slope | 167.4 | 92.7 | - | 225.8 | 29.6 | 23.0 | - | 69.9 | * |
| F slope | 11.0 | 10.5 | - | 11.5 | 9.5 | 8.0 | - | 12.5 |  |
| D slope | 99.7 | 68.3 | - | 106.3 | 94.6 | 69.0 | - | 121.9 |  |
| D/F slope | 8.7 | 7.7 | - | 9.2 | 10.3 | 6.7 | - | 13.4 |  |
| %F slope | 12.0 | 10.4 | - | 13.1 | 10.0 | 8.5 | - | 11.6 | * |
| %D slope | 8.3 | 5.7 | - | 8.9 | 7.9 | 5.7 | - | 10.2 |  |
| L bridge entrance | 222.4 | 156.6 | - | 250.6 | 101.0 | 59.9 | - | 153.3 |  |
| F bridge entrance | 10.0 | 8.5 | - | 11.0 | 7.5 | 5.5 | - | 9.5 |  |
| D bridge entrance | 39.9 | 29.4 | - | 50.6 | 34.7 | 30.6 | - | 63.9 |  |
| D/F bridge entrance | 4.2 | 3.5 | - | 4.8 | 6.0 | 3.5 | - | 8.9 |  |
| %F bridge entrance | 10.7 | 9.3 | - | 12.1 | 7.5 | 5.7 | - | 8.5 | ** |
| %D bridge entrance | 3.3 | 2.5 | - | 4.2 | 2.9 | 2.5 | - | 5.3 |  |
| L bridge | 240.9 | 159.7 | - | 257.1 | 102.0 | 62.9 | - | 154.9 | * |
| F bridge | 5.0 | 4.0 | - | 6.0 | 3.5 | 3.0 | - | 5.0 | * |
| D bridge | 158.0 | 138.2 | - | 202.8 | 104.0 | 87.6 | - | 139.0 | * |
| D/F bridge | 32.0 | 28.7 | - | 46.2 | 30.4 | 26.0 | - | 34.9 |  |
| %F bridge | 5.7 | 4.6 | - | 6.1 | 3.8 | 3.1 | - | 4.0 | ** |
| %D bridge | 13.2 | 11.5 | - | 17.0 | 8.7 | 7.3 | - | 11.6 | * |
| Total activity | 88.5 | 77.0 | - | 99.0 | 95.5 | 86.0 | - | 109.5 | * |
| Distance | 7282.6 | 6363.3 | - | 7883.9 | 7997.1 | 6762.9 | - | 8787.3 |  |
| Velocity | 6.1 | 5.3 | - | 6.6 | 6.7 | 5.7 | - | 7.5 |  |
| Number of visited zones | 10.0 | 10.0 | - | 10.0 | 10.0 | 10.0 | - | 10.0 |  |
| Occ all zones visited | 12/12 |  |  |  | 11/12 |  |  |  |  |
| Rearing | 94.0 | 83.0 | - | 111.5 | 120.0 | 97.0 | - | 128.0 |  |
| Nose pokes | 1.0 | 0.0 | - | 2.0 | 1.0 | 0.0 | - | 2.5 |  |
| Occ nose pokes | 8/12 |  |  |  | 7/12 |  |  |  |  |
| Grooming | 2.0 | 1.0 | - | 2.5 | 2.0 | 1.0 | - | 3.5 |  |
| Occ grooming | 10/12 |  |  |  | 11/12 |  |  |  |  |
| SAP | 0.0 | 0.0 | - | 0.5 | 0.0 | 0.0 | - | 0.0 |  |
| Occ SAP | 3/12 |  |  |  | 0/12 |  |  |  |  |
| Urine | 1.0 | 0.0 | - | 1.0 | 0.0 | 0.0 | - | 1.0 |  |
| Occ urine | 7/12 |  |  |  | 4/12 |  |  |  |  |
| Boli | 0.0 | 0.0 | - | 0.5 | 0.0 | 0.0 | - | 0.0 |  |
| Occ boli | 3/12 |  |  |  | 0/12 |  |  |  |  |
| Occurrence (Occ) is shown for the zones and behaviors that were not visited/performed by all animals in each trial. *p<0.05. **p<0.01 (Wilcoxon matched pairs test). Abbreviations: DCR, dark corner room; D, duration (s); D/F, duration per visit (s); F, frequency; L, latency (s); SAP, stretched attend posture. | | | | | | | | | |
