# Supplementary figures and images for: Adolescent Exploratory Strategies and Behavioral Types in the Multivariate Concentric Square FieldTM Test
Source: Front Behav Neurosci. 2019 Mar 4;13:41. doi: 10.3389/fnbeh.2019.00041 (PMC6409336; doi:10.3389/fnbeh.2019.00041)

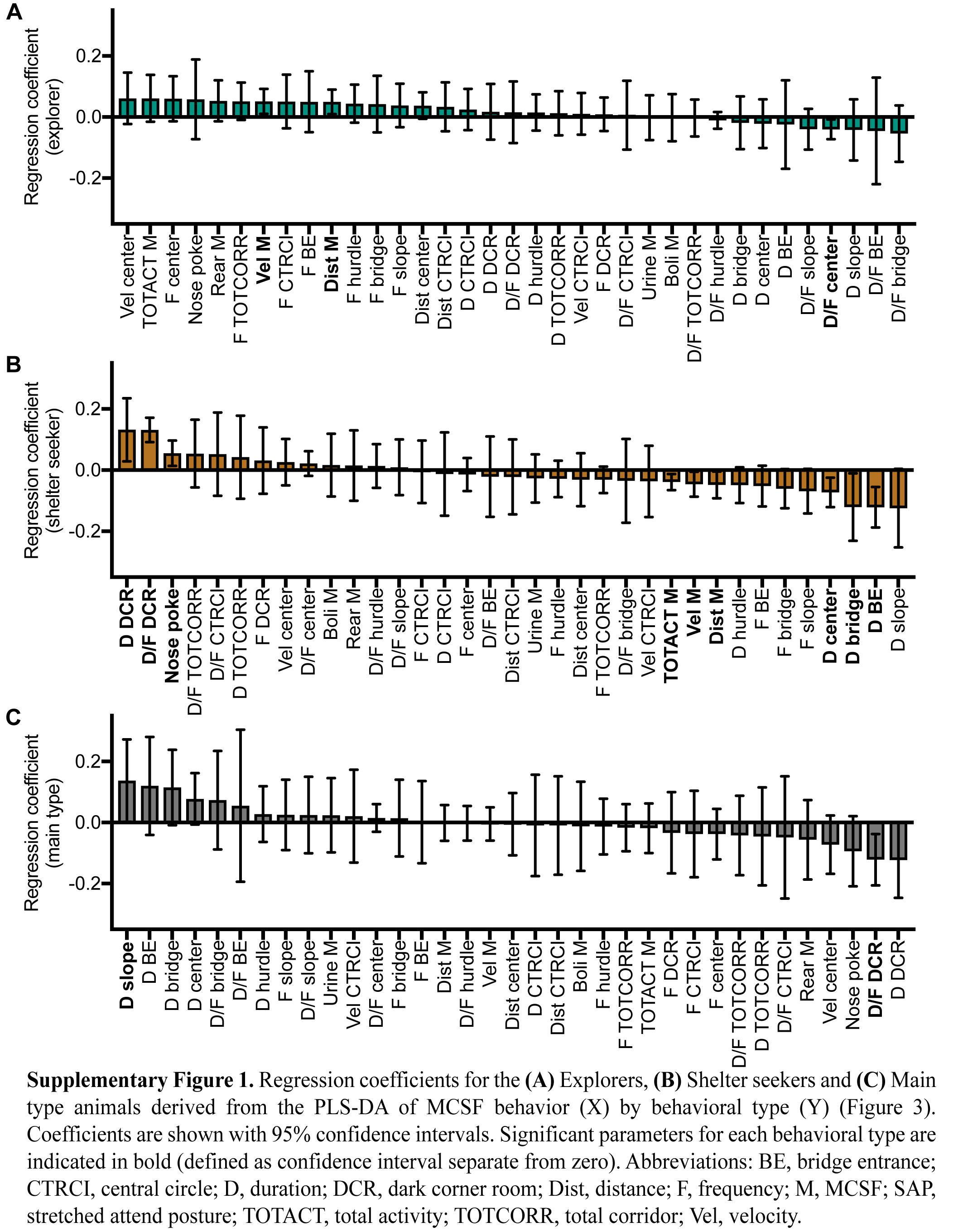

Supplement: Supplementary file 5 [file Image_1.TIF]

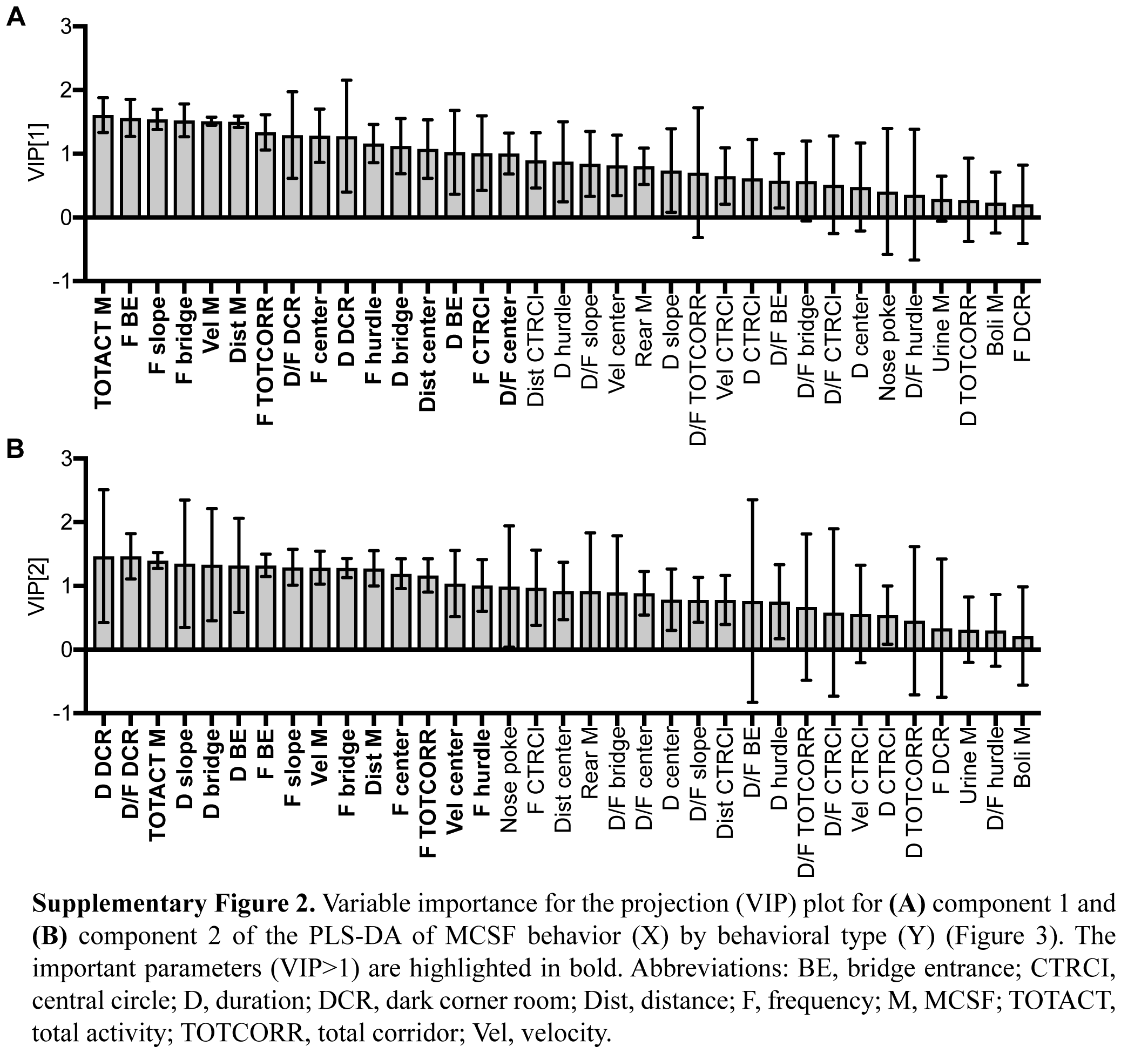

Supplement: Supplementary file 6 [file Image_2.TIF]

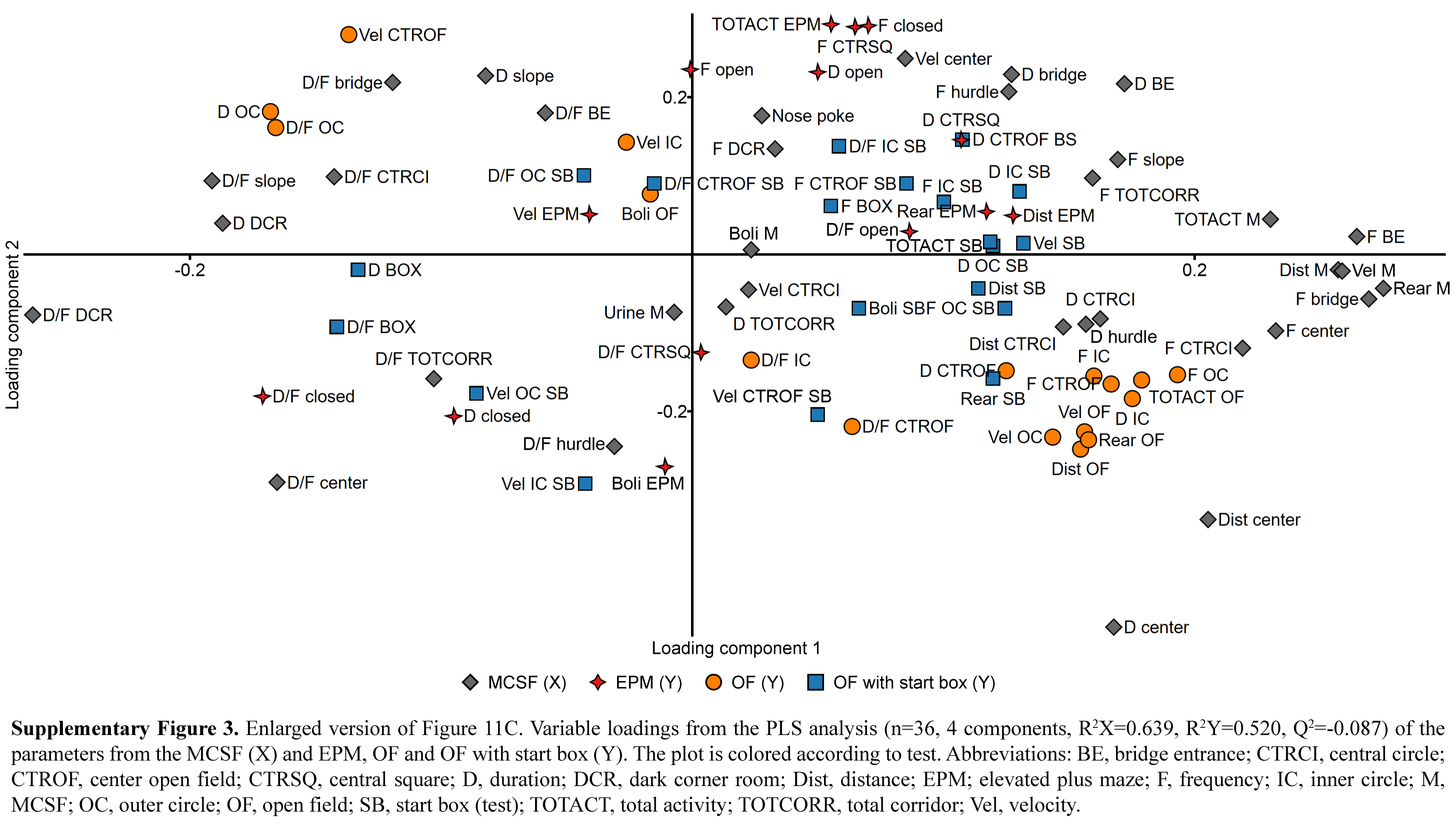

Supplement: Supplementary file 7 [file Image_3.TIF]

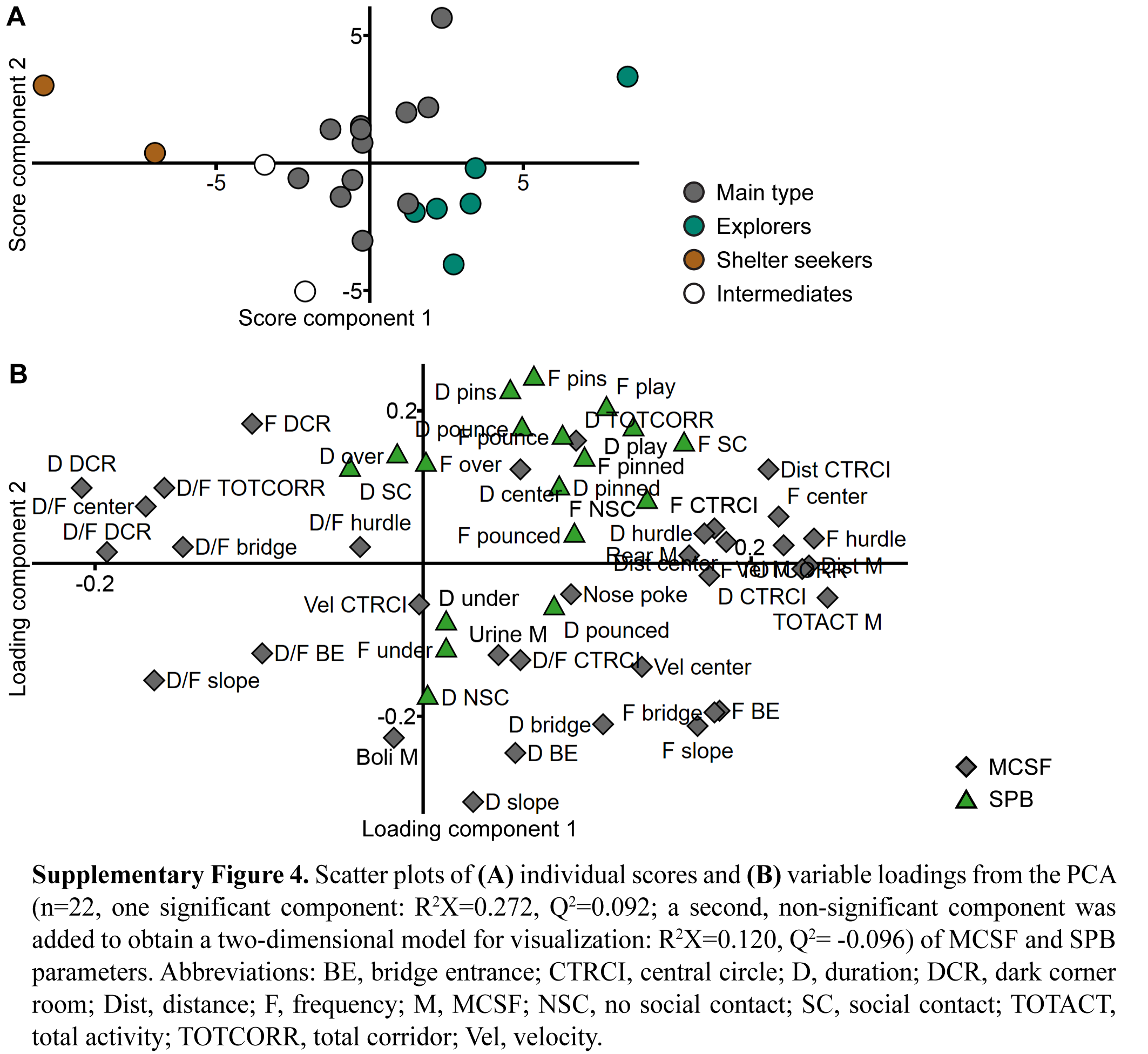

Supplement: Supplementary file 8 [file Image_4.tif]

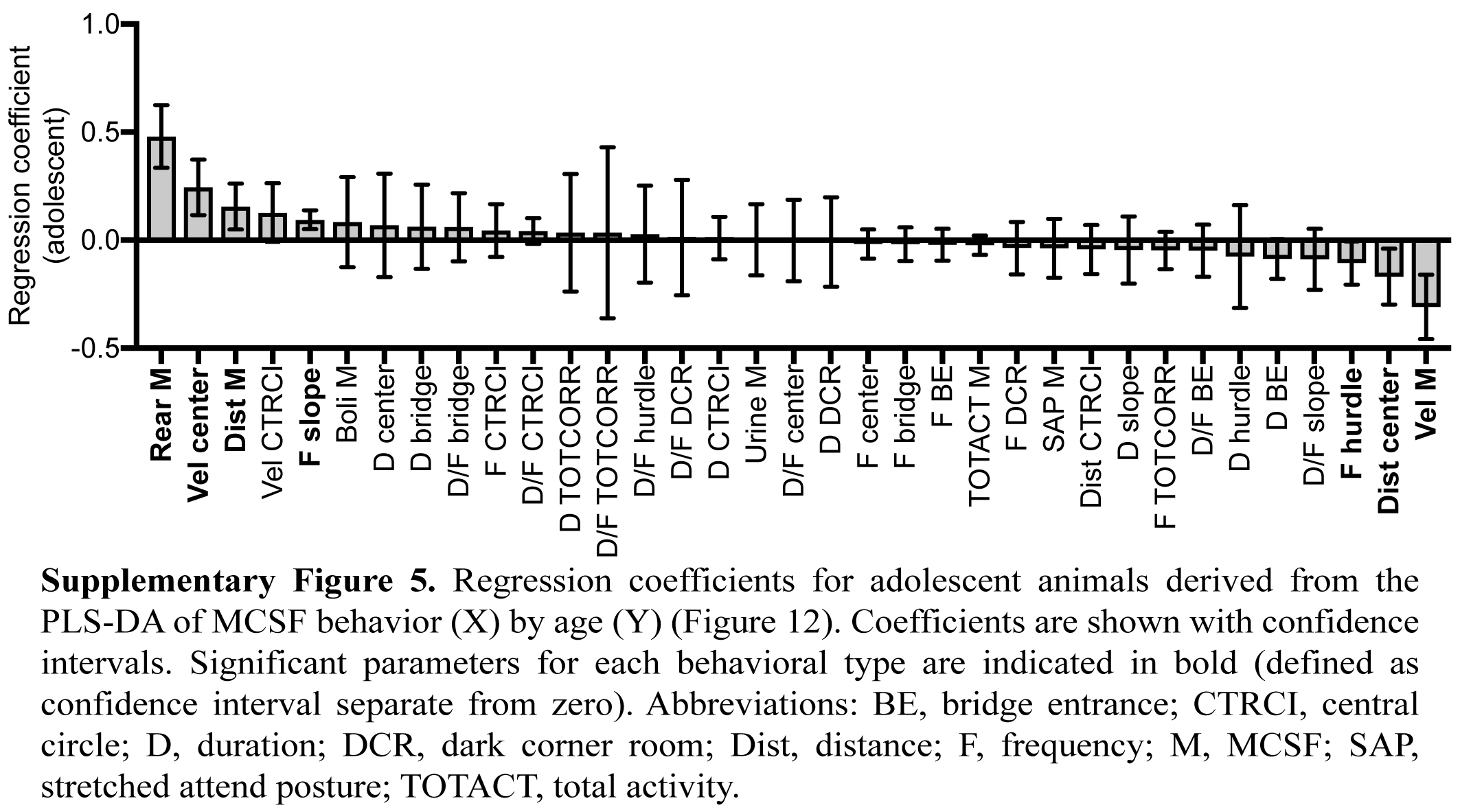

Supplement: Supplementary file 9 [file Image_5.TIF]
